# FOXP1 inhibits pancreatic cancer growth by transcriptionally regulating IRF1 expression

**DOI:** 10.1371/journal.pone.0280794

**Published:** 2023-03-23

**Authors:** Le Wang, Ping Luo, Zhiwen Yang, Xiaoming Zhong, Changxue Ji

**Affiliations:** 1 Graduate School, Medical College, Nanchang University, Nanchang, Jiangxi Province, China; 2 Scientific Research Section, Jiangxi Cancer Hospital, Jiangxi Clinical Research Center for Cancer, Nanchang, Jiangxi Province, China; 3 Department of Breast Surgery, Nanchang Third Hospital, Nanchang, China; 4 Department of Pharmacy, Songjiang District Central Hospital, Shanghai, China; 5 Department of Oncology Radiotherapy, Jiangxi Cancer Hospital, Jiangxi Clinical Research Center for Cancer, Nanchang, Jiangxi Province, China; 6 Department of Vascular Interventional Radiology, Songjiang District Central Hospital, Shanghai, China; Florida International University, UNITED STATES

## Abstract

FOXP1, known as a Forkhead-box (FOX) family protein, plays an important role in human tumorigenesis. However, the function and molecular mechanism of FOXP1 in pancreatic cancer (PC) remain unclear. Here, we report that PC patients with FOXP1 overexpression had a higher survival rate compared to patients with low- FOXP1 expression. Additionally, high expression of FOXP1 can markedly inhibit the growth of pancreatic cancer *in vivo* and *in vitro*, whereas low expression of FOXP1 effectively promoted the tumorigenesis. Mechanistically, FOXP1 could directly bind the IRF1 promoter, which triggered the transcriptional activity of IRF1. Taken together, FOXP1 suppressed PC growth via IRF1-dependent manner, serving as a potential prognostic biomarker for patients with PC.

## Introduction

Pancreatic cancer (PC), as one of the most common gastrointestinal malignancies, is recognized as the fourth leading cause of cancer-related deaths, along with the steadily rising incidence in the worldwide [1,2]. Due to most cases diagnosed at an advanced or distant metastatic stage, patients usually lost the opportunity for surgical resection, and remain the 5-year survival rate at only 6%~8% [3]. Thus, a deeper understanding of the molecular mechanisms related to PC is of clinical significance, as it may not only help in understanding cancer biology, but also provide the base to develop new therapeutic approaches.

FOXP1 known as a Forkhead-box (FOX) family protein has been mapped to chromosome 3p14.1.0 [4]. FOXP1 serves as DNA-binding protein to regulate transcription and DNA repair, which is involved in cell differentiation, growth [5], longevity [6], as well as embryogenesis [7]. Interestingly, FOXP1 plays an important role in the prognosis of cancer patients, acting as a tumor suppressor in some tumors but a cancer driver in others [6,8]. For instance, FOXP1 upregulation reveals a poor outcome in diffuse large B-cell lymphoma [9], gastric mucosa-associated lymphoid tissue lymphoma [10] and hepatocellular carcinoma [11], but a good prognosis in breast cancer [12].

In this study, our aim was to explore the biological function and clinical significance of FOXP1 in PC. FOXP1 upregulation can inhibit the growth of PC, showing a better overall survival of PC patients. Of note, FOXP1 is identified as a transcriptional target of IRF1. Thereby, FOXP1 effectively suppressed the PC progression via IRF1-dependent manner, providing a potential therapeutic target.

## Methods

### Tissue microarrays (TMAs) and immunohistochemistry (IHC)

Human formalin fixed paraffin embedded pancreatic cancer tissue microarray slides were obtained from Zhuo li biotechnology Co., Ltd (Shanghai, China). Briefly, tumor and adjacent normal tissues of PC patients were collected from January 2012 to December 2016. The average age of patients was 62.8 ± 10.1 y, ranging from 34 to 79 years. Following-up data from all patients were collected. This study has been approved by the Medical Ethics Committee of our institution.

TMAs were used to perform the clinic pathological assessment. FOXP1 expression was detected with IHC. A total of eight samples were absent in the array. A final score from IHC parameters was built according to the following criteria [13]: negative scores had a staining intensity of 0 and 1+ in ≤10% of tumor cells; Positive scores were defined as at least weak expression in tumor.

### Cell culture

The HPDE, CFPAC1, SW1990, PANC1, and CAPAN1 cell lines were obtained from Chinese Academy of Sciences (Shanghai, China). All pancreatic cancer cells were cultured in RPMI Medium 1640 (Gibco, USA) containing 10% fetal calf serum (Gibco, USA), penicillin (100 unites/ml), streptomycin (100 μg/ml), and maintained at 37°C in a humidified atmosphere with 5% CO2.

### Plasmids, small interfering RNAs and transfection

**Fig 1:**
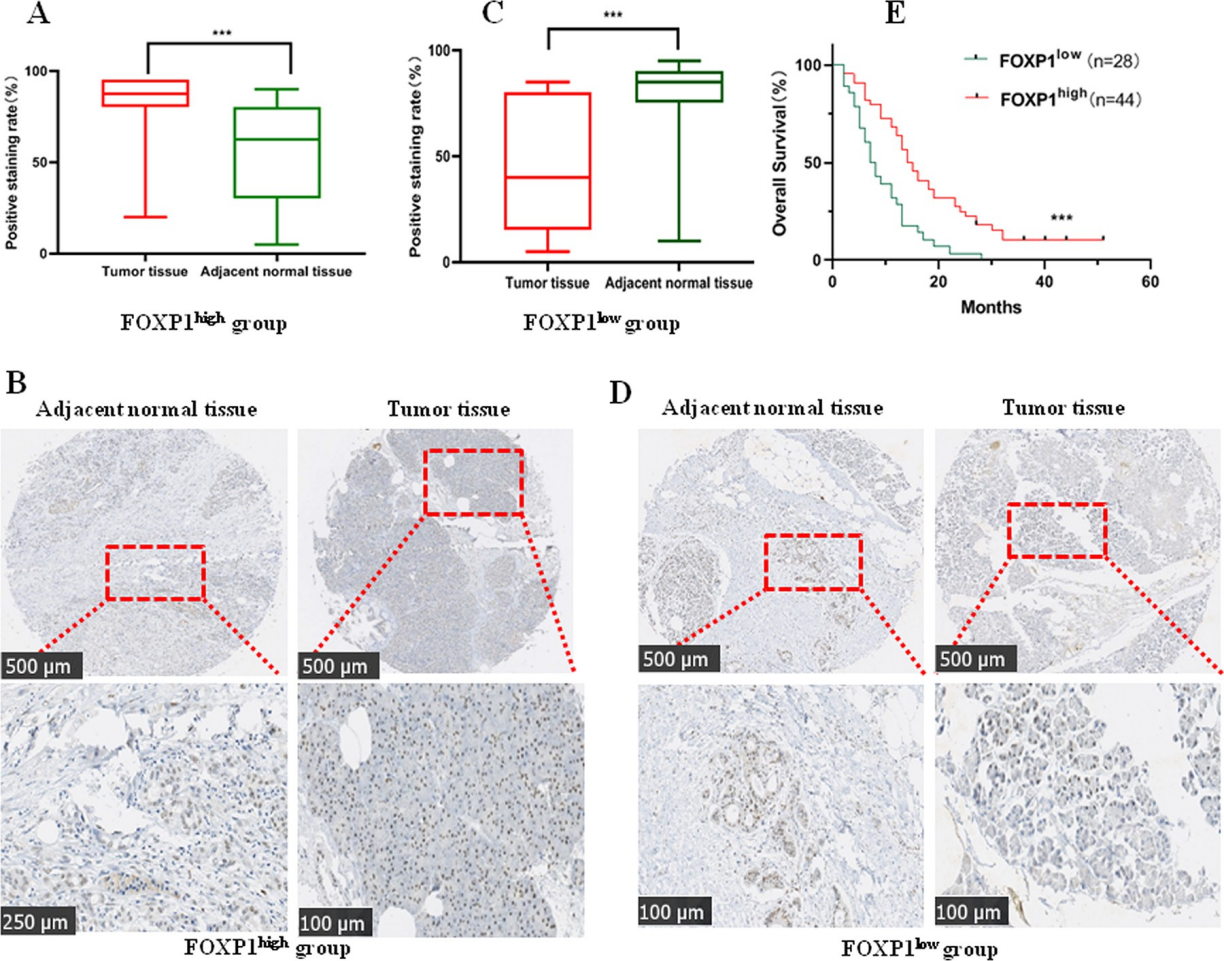
FOXP1 was responsible for a good prognosis in PC patients. A-B: FOXP1^high^ expression in 44 PC patients; C-D: FOXP1^low^ expression in 28 PC patients; E: Kaplan-Meier methods analyzed the survival rates of 72 PC patients with FOXP1^high^ and FOXP1^low^ expression. The expression of FOXP1 was determined by an in-situ hybridization in 72 pairs of pancreatic tumor and matched adjacent non-tumor tissues. *P<0.05; **P<0.01 ***P<0.001.

Expression vectors encoding FOXP1 were constructed and inserted into the pcDNA3.1 vector (oeFOXP1), while the empty pcDNA3.1 vectors were used as negative control (Vector). pcDNA3.1 (+) vector for FOXP1 overexpression, and their corresponding negative controls were all synthesized by GenePharma (Shanghai, China). Additionally, oeFOXP1 were transfected into PANC1 cells. Briefly, cells were seeded on a six-well plate at a density of 5×10^5^ cells/well. Transfection operation began after 24 h incubation at 37°C in a humidified incubator. After 48 h transfection, the medium was replaced by fresh medium containing 0.5 mg/ml G418 for screening the stable expression of FOXP1 (Fig 1).

The siRNA sequence targeting FOXP1 was synthesized by GenePharma (Shanghai, China). The siRNA sequence targeting FOXP1 (siFOXP1) was 5’- GCAGUUAGAGCUACAGCUUTT -3’. A nonspecific scrambled siRNA sequence (si-scramble) was 5’-CAGUACUUUUGUGUAGUACAA-3’, serving as negative control. siFOXP1 were respectively transfected into PANC1 and CFPAC1 cells according to the manufacture instructions.

### Real-time polymerase chain reaction (RT-PCR)

RT-PCR was applied to detect the expression of FOXP1 mRNA. Briefly, total RNAs were extracted from PC cells by using TRIZOL reagent (Yeasen, Shanghai, China). Then, RNA was reversely transcribed into cDNAs, and GAPDH was used as the internal control. Each experiment was repeated in triplicate. Fold change was calculated by 2^–△△Ct^ methods.

### Western blot

The whole-cell lysates were extracted by RIPA buffer mixture (Beyotime, Shanghai). Protein was separated in 10% SDS-PAGE, and then transferred to PVDF membrane. The membrane was respectively incubated with FOXP1 and IRF1 primary antibodies (CST, USA). GAPDH acted as an endogenous protein for normalization.

### Cell viability

The cell viability was determined using the cell counting Kit-8 (CCK-8) kit. Briefly, 1 × 10^4^ cells were seeded into 96-well plates and inoculated at the indicated times (the 1^st^, 2^nd^, and 3^rd^ day). 10 μl of CCK-8 solution was added into each well for 3 h incubation, and the absorbance at 450 nm was detected by using a spectrophotometer (Thermo, Waltham, MA, USA).

### Transwell assay

The transwell assay was performed by using 24-well transwell plates (8 μm pore size, Corning, NY, USA) with a matrigel-coated membrane for the invasion assays. Cells were seeded in the upper compartment, and RPMI-1640 culture medium was added into the lower compartment. After 24 h incubation, cells were fixed with 4% paraformaldehyde, and stained with 1% crystal violet.

### Clone formation assay

The clone formation assay was performed using 6-well plates in a density of 500 cells/well. The cells were allowed to grow for 21 days, and cell colonies were counted by using an inverted microscope.

### Wound healing assay

The clone formation assay was performed by using 6-well plates in a density of 3×10^5^ cells/ml. At 0, 12 and 24 h after scratch, wound images were captured through the measurement of wound width.

### Luciferase reporter assay

Luciferase reporter assay was detected by using the Dual-Luciferase Reporter Assay kit (Promega, Madison, WI). After 48 h incubation, the transfected cells were lysed and assayed for luciferase activity with a dual-luciferase reporter assay system. In brief, 293T cells were transiently transfected with the different pGL3-Enhancer-wtIRF1 plasmids (Genepharma, Shanghai) together with FOXP1 siRNA or FOXP1-expressing plasmid. pRL-TK vector was used to serve as internal control.

### Chromatin immunoprecipitation assay (ChIP)

ChIP assay was performed by using a ChIP assay kit (Upstate Biotechnology, MA, USA) following manufacturer’s instructions. Briefly, cell was lysed, and chromatin was sonicated. DNA-protein complexes were immunoprecipitated with FOXP1 and IRF1 antibodies. Mouse immunoglobulin G (IgG) acted as a negative control. After removing RNA and protein, the ChIP-derived DNA samples were subjected to polymerase chain reaction. The primers were listed in below: IRF1-F, 5’TCTTCCCATCACAGCAAACC3’, and R, 5’AGCGCTCCCAATCCACC3’.

### Tumor growth assays *in vivo*

BALB/C nude mice were obtained from the Shanghai Experimental Animal Centre (Shanghai, China). The protocol was approved by the Institutional Animal Care and Use Committee of Shanghai Rat & Mouse Biotech Co.,Ltd. Male Balb/c nude mice (4–6 weeks old, 20±2 g) were kept under specific pathogen-free conditions. 2×10^6^ PANC1 cells transduced with empty vector or FOXP1 overexpressing vector (oeFOXP1) were injected subcutaneously into left axilla of each nude mouse (6 mice per group). Once tumors became palpable, growing xenografts were measured with a caliper every three days. The volume of tumor was calculated using the formula: V (mm3) = 1/2×length×width^2^. Mice for signs of pain were monitored during the study. If necessary, buprenorphine was used to lessen their distress. All mice were euthanized with anaesthetic pentobarbital sodium by intraperitoneal injection, and then sacrificed 33 days after inoculation. The tumor were harvested, imaged and weighed.

### Statistical analysis

All data were processed with SPSS version 20 software and presented as mean ± standard deviation for multiple. Kaplan-Meier method was carried out to estimate the survival rates. Student *t* test or One-way analysis was used for parametric variables. Mann-Whitney was performed for non-parametric variables. *p*<0.05 was considered as statistically significant.

## Results

### FOXP1 is responsible for a good prognosis in PC patients

To assess the expression level of FOXP1 in PC, immunohistochemistry was applied to assay FOXP1 expression in 72 paired samples. Of note, FOXP1 showed an obvious increase in PC tissues of 44 patients related to their matched normal pancreatic tissues (Fig 1A and 1B), serving as FOXP1^high^ group. A decrease in PC tissues of 28 patients was found as compared to their normal tissues, acting as FOXP1^low^ group (Fig 1C and 1D). After comparing the average expression of FOXP1 between tumor and normal tissue, an obvious increase in PC tissues is found as compared to the normal pancreatic tissues (*P* = 0.046). Then, Kaplan-Meier analysis was applied to determine the relationship between FOXP1 expression and clinicopathological characteristics, indicating a better prognosis in PC patients with the FOXP1^high^ group (Fig 1E). Thus, the high expression of FOXP1 is associated with a good prognosis in PC patients.

### FOXP1 inhibits tumor growth *in vivo*

**Fig 2:**
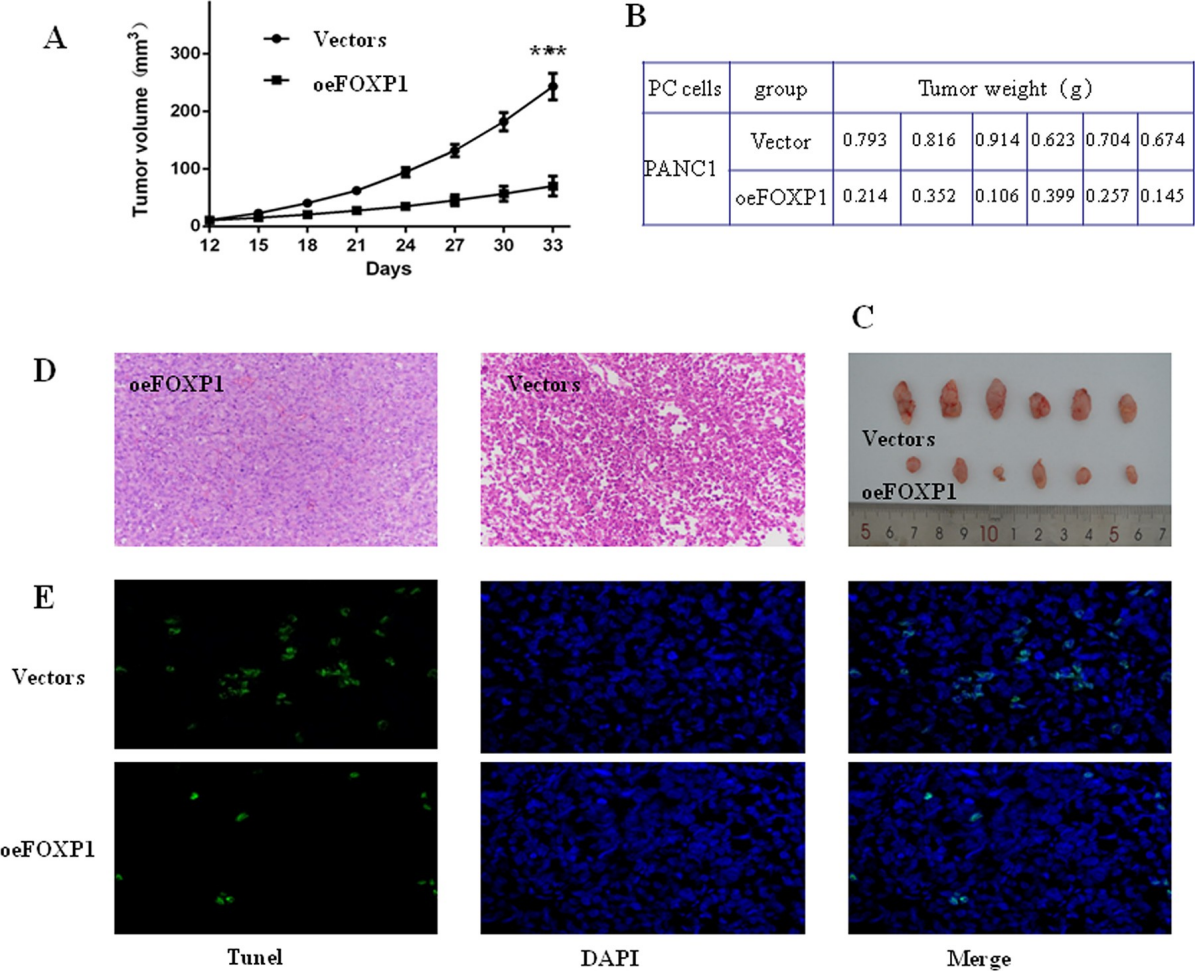
FOXP1 inhibited the growth of pancreatic cancer *in vivo*. A: Tumors growth volume; B and C: Tumor weight at 33 days (vector vs. oeFOXP1, *p*<0.0001); D: Histopathology (magnification ×200); E: TUNEL stain (magnification ×400). *P<0.05; **P<0.01 ***P<0.001.

To further investigate the effect of FOXP1 on the tumor growth, a subcutaneous tumor xenograft model in nude mice was established by injecting FOXP1-upregulated PANC1 cells. After 21 days, the oeFOXP1 group had a dramatically decreased tumor growth compared with the control group (27.4±10.3mm^3^ vs. 62.2±10.7mm^3^, p<0.01). On day 33, tumors growth between these two groups reached a maximum contrast (Fig 2A). Consistent with tumor volume, the tumor weights (Fig 2B and 2C) further demonstrated that FOXP1 expression could hinder tumor growth. Histopathology in high-FOXP1 expression was indicative of the massive cancer cell remission, including tumor nucleus fragmentation, deformation, cell disorder arrangement, coagulative necrosis and intercellular blank *etc* (Fig 2D). As shown in Fig 2E, lower level of immunofluorescence was found in oeFOXP1 group, suggesting that FOXP1 significantly decreased the DNA damage in tissues. Together, high-expressed FOXP1 efficiently inhibit the growth of pancreatic cancer *in vivo*.

### FOXP1 inhibits the growth *in vitro*

**Fig 3:**
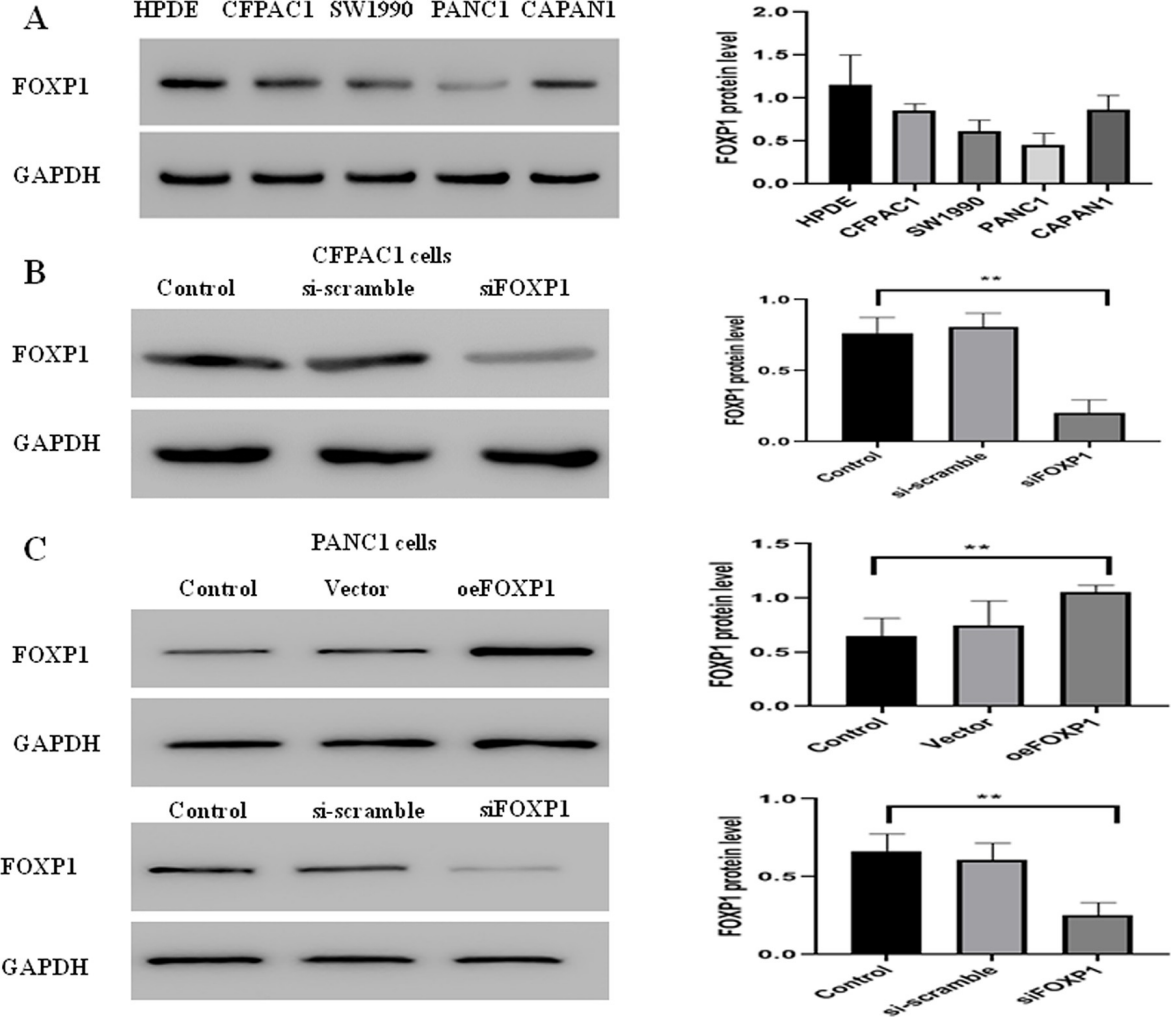
The expression level of FOXP1 protein in pancreatic cancer cells. A: FOXP1 protein level in five pancreatic cancer cell lines; B: FOXP1 protein level in CFPAC1 after siFOXP1 (control vs. siFOXP1, *p* = 0.0025); C: FOXP1 protein level in PANC1 after siFOXP1 and oeFOXP1 (control vs. siFOXP1, *p* = 0.0064; control vs. oeFOXP1, *p* = 0.0162). *P<0.05; **P<0.05; ***P<0.001.

The expression level of FOXP1 protein was assayed in five pancreatic cancer cell lines, indicating a slight expression in PANC1 cells but an obvious increase in the other four cells (Fig 3A).

**Fig 4:**
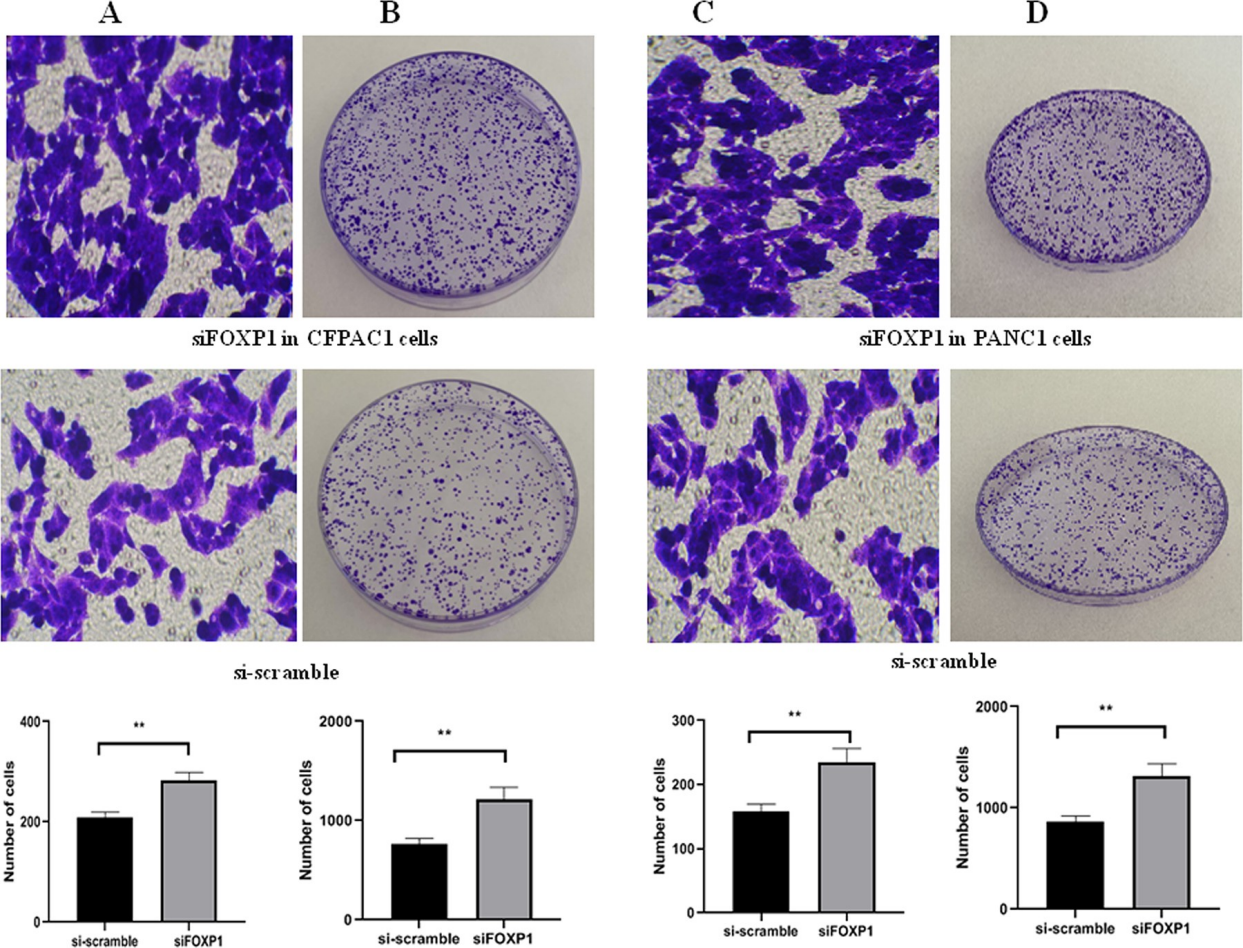
Low-FOXP1 expression promoted the proliferation, migration, and invasion of PANC1 and CFPAC1 cells. A: Transwell assay in CFPAC1 cells (si-scramble vs. siFOXP1, *p* = 0.0032); B: Colony formation assay in CFPAC1 cells (si-scramble vs. siFOXP1, *p* = 0.0040); C: Transwell assay in PANC1 cells (si-scramble vs. siFOXP1, *p* = 0.0049); D: Colony formation assay in PANC1 cells (si-scramble vs. siFOXP1, *p* = 0.0040). Transwell assays were detected after incubation at 37°C for 24 h. Colony formation assays were detected after incubation at 37°C for 21 days. *P<0.05; **P<0.05; ***P<0.001.

**Fig 5:**
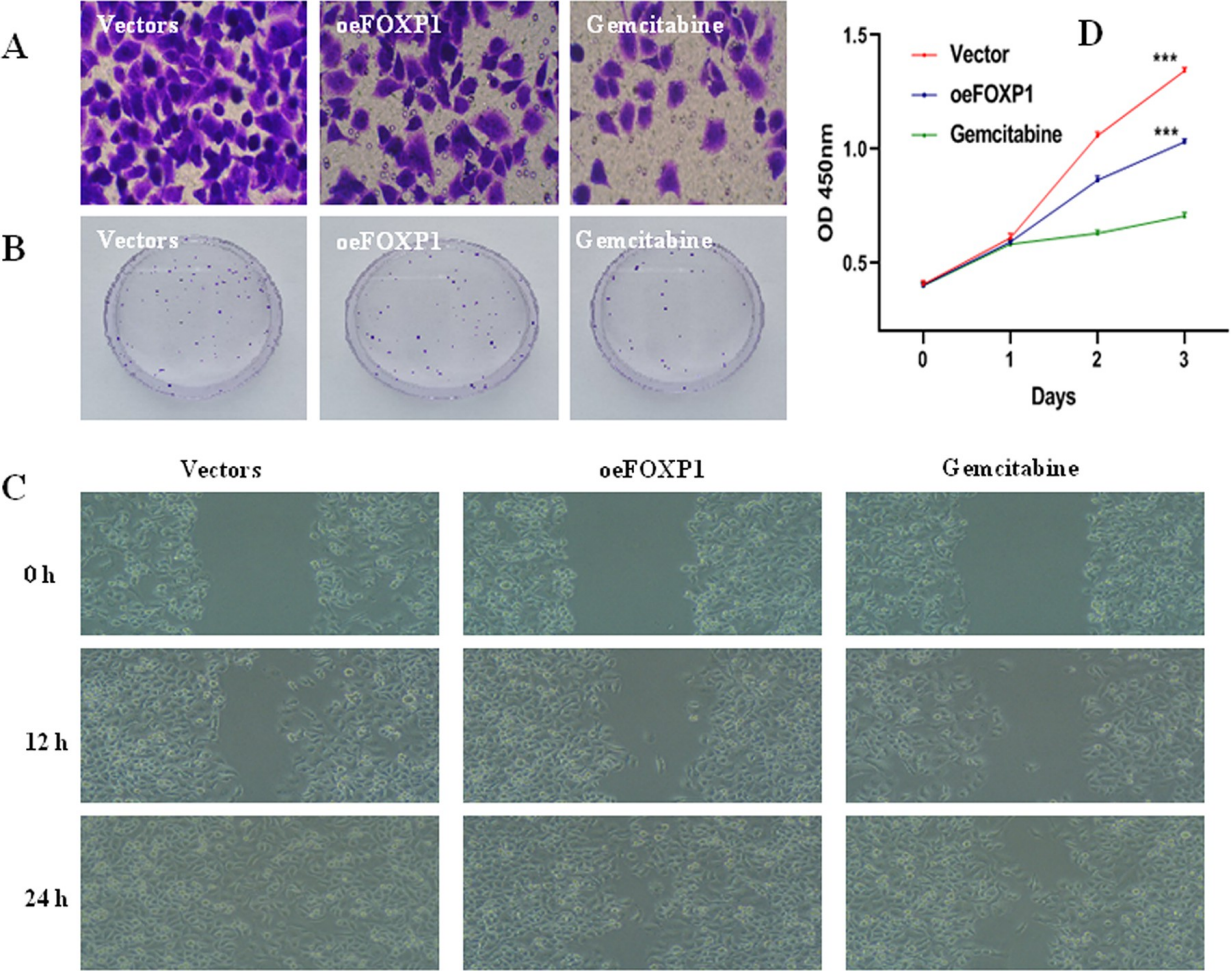
High-FOXP1 expression inhibited the proliferation, migration, and invasion of PANC1 cell. A: Transwell assay; B: Colony formation assay (107±5 in vectors vs 80±8 in oeFOXP1, *p* = 0.0058); C: Wound healing assay (185±27 in vectors at 24h vs 274±18 in oeFOXP1 at 24h, *p* = 0.0088); D: CCK8 assays (1.34±0.01 in vectors vs 1.03±0.01 in oeFOXP1, *p*<0.0001; 1.34±0.01 in vectors vs 0.70±0.01 in gemcitabine, *p*<0.0001). Transwell assay were detected after incubation at 37°C for 24 h. Colony formation assays were detected after incubation at 37°C for 21days. Wound healing assays were detected after incubation at 37°C for 0, 12, 24 h. CCK8 assays were detected after incubation at 37°C for 0, 24, 48, 72 h, and PANC1 cell was treated with 5 μmol/L gemcitabine. *P<0.05; **P<0.05; ***P<0.001.

To confirm the role of FOXP1, the siRNA knockdown of FOXP1 was performed in CFPAC1 cell lines. Western blot exhibited a dramatically decreased expression of FOXP1 after siFOXP1 knockdown (Fig 3B). Transwell assay (Fig 4A) and colony formation assay (Fig 4B) validated that the silenced FOXP1 expression could effectively boost the invasion and proliferation of CFPAC1 cells. Next, the siRNA knockdown and overexpression of FOXP1 was performed in PANC1 cell lines by pcDNA3.1-FOXP1 and siFOXP1 (Fig 3C). Transwell assay (Fig 5A) and colony formation assay (Fig 5B) exhibited that high-FOXP1 expression inhibited the invasion and proliferation of PANC1 cell. CCK-8 assay showed that cell viability in PANC1 cells was obviously decreased after FOXP1 overexpression (Fig 5D). Wound healing assay indicated a significant inhibition of cellular migration by FOXP1 and gemcitabine (Fig 5C). Conversely, after silencing the FOXP1 expression, transwell assay (Fig 4C) and colony formation assay (Fig 4D) showed that the low expression of FOXP1 could promote the growth of PANC1 cells. Together, FOXP1 positively inhibit the development and progression of pancreatic cancer cells.

### FOXP1 is associated with IRF1 expression

**Fig 6:**
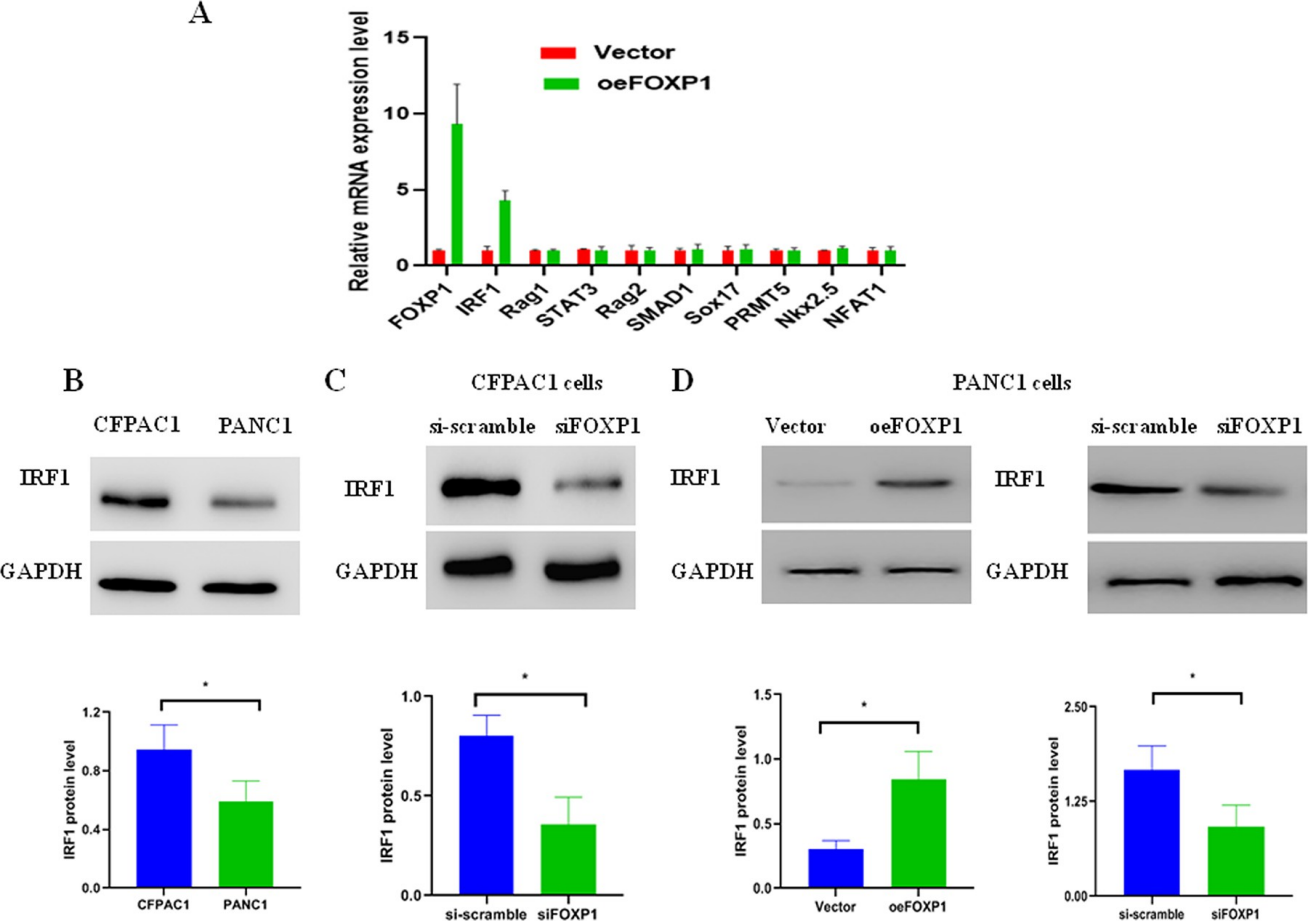
FOXP1 expression level was a close correlation with IRF1. A: Identification of significantly affected genes by FOXP1 in pancreatic cancer cell; B: IRF1 expression level in CFPAC1 and PANC1 cell lines (*p* = 0.0484); C: IRF1 protein level in CFPAC1 after siFOXP1 transfection (si-scramble vs. siFOXP1, *p* = 0.0103); D: IRF1 protein level in PANC1 increased after oeFOXP1 siFOXP1 and transfection (vector vs. oeFOXP1, *p* = 0.0156; si-scramble vs. siFOXP1, *p* = 0.0404).

To identify the FOXP1 downstream targets in pancreatic cancer, 10 previously reported genes, that are regulated by FOXP1 and simultaneously contribute to breast, liver, and ovarian cancer, were tested [4,6,14]. RT-PCR was applied to assess the alterations of mRNA levels of 10 genes between the oeFOXP1 and control group. The IRF1 mRNA was obviously increased in the oeFOXP1 group, but not the other gene mRNA (Fig 6A). This result indicated that FOXP1 may be responsible for IRF1 regulation in pancreatic cancer.

To further determine the correlation between IRF1 and FOXP1, CFPAC1 (high-FOXP1 expression) and PANC1 cell lines (low-FOXP1 expression) were selected. Firstly, the expression level of IRF1 was determined in CFPAC1 and PANC1 cell lines, respectively. IRF1 high expression was found in CFPAC1, but low expression in PANC1 (Fig 6B). Second, the FOXP1 was knockout in CFPAC1. The result showed a decrease in IRF1 expression along with FOXP1 knockout (Fig 6C). Third, the expression level of FOXP1 was increased and/or decreased in PANC1 cell lines. IRF1 protein level was upregulated in the oeFOXP1, but downregulated in the siFOXP1 (Fig 6D). These data confirmed a close correlation between IRF1 and FOXP1.

Additionally, after indexing the transcription factor database, FOXP1 binding site was likely located in the region of GGTTGAAAAACAGAG in the IRF1 promoter (S1 and S2 Tables). Thereby, FOXP1 has a potential ability to bind to the IRF1 promoter region in pancreatic cancer.

### FOXP1 increases IRF1 transcription activity

**Fig 7:**
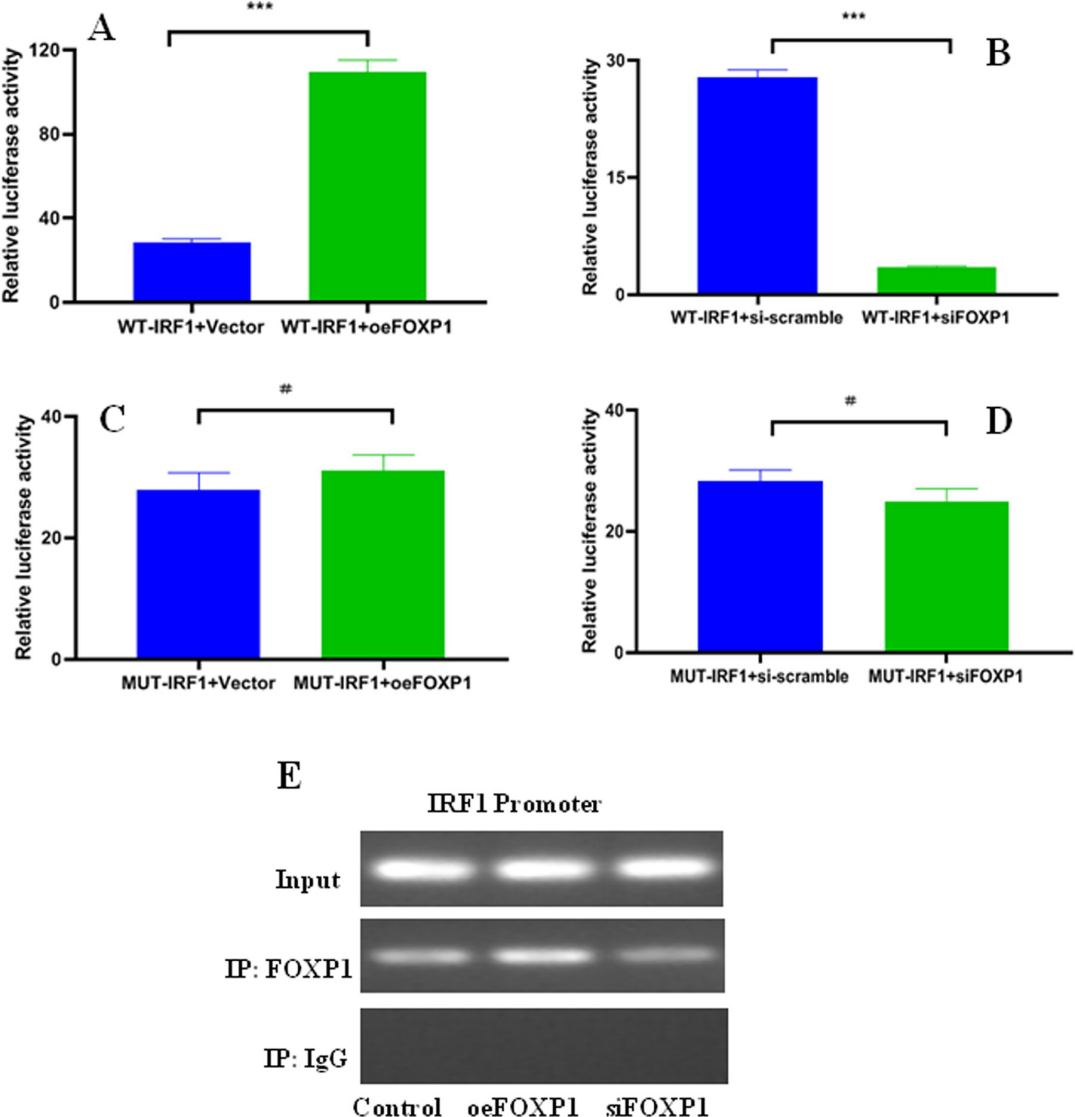
FOXP1 directly bound to the IRF1 promoter region. A: IRF1 promoter activity in oeFOXP1 and WT-IRF1 (*p*<0.0001); B: IRF1 promoter activity in siFOXP1 and WT-IRF1 (*p*<0.0001); C: IRF1 promoter activity in oeFOXP1 and MUT-IRF1 (*p* = 0.2253); D: IRF1 promoter activity in siFOXP1 and MUT-IRF1 (*p* = 0.1146). E: FOXP1 could directly bind to the IRF1 specific promoter region. The relative luciferase activity and ChIP assays performed in 293T cells. ^#^*P>0*.05; *P<0.05; **P<0.05; ***P<0.001.

To determine the IRF1 transcription mediated by FOXP1, dual luciferase reporter assays were enforced by generating pGL3-Enhancer-wtIRF1 and internal control plasmid pRL-TK. The relative luciferase activity exhibited an obvious increase in oeFOXP1 group (Fig 7A), but an obvious decrease in siFOXP1 group (Fig 7B). Thus, FOXP1 may be involved in regulating IRF1 transcription activity.

### FOXP1 binds to the IRF1 promoter region

To confirm the FOXP1 binding sites in the promoter region of the IRF1 gene, pGL3-Enhancer-mutIRF1 and ChIP assays was preformed. The relative luciferase activity in mutIRF1 did not show a significant boost and/or reduce after oeFOXP1 and siFOXP1 (Fig 7C and 7D), revealing a potential binding site between FOXP1 and IRF1. ChIP assays demonstrated that anti-Flag antibody could effectively access and capture the binding site of FOXP1, but not isotype IgG (Fig 7E). The results verified the physical binding of FOXP1 to IRF1 promoter. Therefore, FOXP1 could directly bind to the IRF1 specific promoter region (S1 and S2 Tables) and effectively regulate IRF1 transcription.

## Discussion

In the present study, we found that FOXP1 expression level in 44 PC tissues was obviously elevated, but not in 28 PC tissues. Interestingly, PC patients with FOXP1 overexpression exhibited a better prognosis than those patients with FOXP1 downregulation. Of note, FOXP1-bound IRF1 promoter was found by luciferase and ChIP assays, suggesting a possibly of elevating the expression level of IRF1 that inhibited tumor cell growth and invasion. Therefore, we concluded that FOXP1 served as a tumor suppressor in PC progression.

Emerging research showed the dual functions of FOXP1 protein in specific cancer types. FOXP1 was known as a tumor promoter with a poor prognosis in follicular lymphoma [15], primary cutaneous large B-cell lymphomas (PCLBCL) [16,17], gastric mucosa-associated lymphoid tissue lymphoma (MALT) [10] and diffuse large B-cell lymphoma (DLBCL) [9,18–20]. The signaling pathways maybe attribute to the G1/S phase arrest and decreased phosphorylation of retinoblastoma protein [21], suppression of MHC class II expression and activation of Wnt/βcatenin signaling [22,23], interaction between FOXP1 and estrogen receptor beta or alpha in the nuclear [12,24], and activation of chromosome translocations via immunoglobulin heavy chain enhancers [11,25]. In contrary, FOXP1 worked also as a tumor suppressor with good prognosis in breast cancer [8] and lung carcinoma [26]. Mechanistically, FOXP1 gene generally mapped to a tumor suppressor locus at 3p14.1, repressed AR-induced transcriptional activity or histone modification [27], and interacted between FOXP1 and NFAT1 [28].

To date, it is still unclear whether FOXP1 protein works as an oncogenic or tumor suppressive role in PC. In a previous study, FOXP1 provoked the formation of multiple polyploid lesions in PC patients [29]. FOXP1 knockdown can reduce the expression of N-cadherin, displaying a reversed epithelial morphology [29]. However, our study found that FOXP1 acted as a tumor suppressor in PC progression. FOXP1 upregulation inhibited tumor cell growth, whereas FOXP1 knockdown had a opposite result. The discrepancy may be attribute to the dual role of FOXP1 in PC, tumor-promoting and tumor-suppressive function.

FOXP1, as a transcription factor, consists of a FOX domain, a leucine zipper domain, a C2H2-type zinc finger domain, and a poly-Gln region [30]. FOXP1 regulates the expression and transcriptional activity of many genes, which are involved in different stages of tumor, such as initiation, promotion and progression. To determine the FOXP1 downstream targets, we screened a series of genes that are critically important for tumor progression and highly correlated with FOXP1 expression [4,6,14]. IRF1 in PANC1 cell lines was markedly upregulated by FOXP1, indicating a positive correlation between IRF1 and FOXP1.

Originally, IRF1 is thought to be a transcription factor that triggered the expression of β-interferon. With more in-depth research, IRF1 could also regulate the expression of target genes via activating the tumor suppressor p53 [31], binding with its cofactor P300 [31] or interferon stimulated response elements [32,33], and processing antigens for presentation by cytotoxic T cells and repairing the major histocompatibility complex I [34]. It has been established that IRF1 works as a tumor suppressor in various tumor, including gastric cancer, esophageal cancer, breast cancer, and renal cell carcinoma [35,36]. Notably, we found that FOXP1 could directly bind to the IRF1 specific promoter region. Collectively, FOXP1 as a tumor suppressor inhibited PC progression by triggering the transcriptional activity of IRF1.

In summary, we unveiled that FOXP1 acted as a tumor suppressor in PC progression. Mechanistically, FOXP1 directly bound to the IRF1 promoter region, enhancing IRF1 transcriptional activity.

## Supporting information

S1 Fig FOXP1 expression level in PANC1 cell after stable transfection. (TIF)

S1 Table wtIRF1 promoter. (DOC)

S2 Table mutIRF1 promoter. (DOC)

S1 File (DOCX)

## References

[1] Guo X, Li K, Jiang W, Hu Y, Xiao W, Huang Y, et al. RNA demethylase ALKBH5 prevents pancreatic cancer progression by posttranscriptional activation of PER1 in an m6A-YTHDF2-dependent manner. *Mol cancer*. 2020; 19(1):91. doi: 10.1186/s12943-020-01158-w 32429928 PMC7236181

[2] Liang C, Shi S, Qin Y, Meng Q, Hua J, Hu Q, et al. Localisation of PGK1 determines metabolic phenotype to balance metastasis and proliferation in patients with SMAD4-negative pancreatic cancer. *Gut*. 2020, 69(5):888–900. doi: 10.1136/gutjnl-2018-317163 31611300

[3] Mizrahi JD, Surana R, Valle JW, Shroff RT. Pancreatic cancer. *Lancet* 2020; 395(10242):2008–2020. doi: 10.1016/S0140-6736(20)30974-0 32593337

[4] Kim JH, Hwang J, Jung JH, Lee HJ, Lee DY, Kim SH. Molecular networks of FOXP family: dual biologic functions, interplay with other molecules and clinical implications in cancer progression. *Mol cancer*. 2019; 18(1):180. doi: 10.1186/s12943-019-1110-3 31815635 PMC6900861

[5] Liu XM, Du SL, Miao R, Wang LF, Zhong JC. Targeting the forkhead box protein P1 pathway as a novel therapeutic approach for cardiovascular diseases. *Heart Fail Rev*. 2022;27(1):345–355. doi: 10.1007/s10741-020-09992-2 32648149

[6] Katoh M, Igarashi M, Fukuda H, Nakagama H, Katoh M. Cancer genetics and genomics of human FOX family genes. *Cancer Lett*. 2013;328(2):198–206. doi: 10.1016/j.canlet.2012.09.017 23022474

[7] Morikawa Y, Komori T, Hisaoka T, Senba E. Detailed expression pattern of Foxp1 and its possible roles in neurons of the spinal cord during embryogenesis. D*ev Neurosci*. 2009;31(6):511–22. doi: 10.1159/000243715 19797899

[8] Koon HB, Ippolito GC, Banham AH, Tucker PW. FOXP1: a potential therapeutic target in cancer. *Expert Opin Ther Targets*. 2007;11(7):955–65. doi: 10.1517/14728222.11.7.955 17614763 PMC4282158

[9] Barrans SL, Fenton JA, Banham A, Owen RG, Jack AS. Strong expression of FOXP1 identifies a distinct subset of diffuse large B-cell lymphoma (DLBCL) patients with poor outcome. *Blood* 2004, 104(9):2933–2935. doi: 10.1182/blood-2004-03-1209 15238418

[10] Han SL, Wu XL, Wan L, Zeng QQ, Li JL, Liu Z. FOXP1 expression predicts polymorphic histology and poor prognosis in gastric mucosa-associated lymphoid tissue lymphomas. *Dig Surg*. 2009;26(2):156–62. doi: 10.1159/000212058 19365123

[11] Zhang Y, Zhang S, Wang X, Liu J, Yang L, He S, et al. Prognostic significance of FOXP1 as an oncogene in hepatocellular carcinoma. *J Clin Pathol*. 2012;65(6):528–33. doi: 10.1136/jclinpath-2011-200547 22422806

[12] Fox SB, Brown P, Han C, Ashe S, Leek RD, Harris AL, et al. Expression of the forkhead transcription factor FOXP1 is associated with estrogen receptor alpha and improved survival in primary human breast carcinomas. *Clin Cancer Res*. 2004;10(10):3521–7. doi: 10.1158/1078-0432.CCR-03-0461 15161711

[13] Burdelski C, Jakani-Karimi N, Jacobsen F, Moller-Koop C, Minner S, Simon R, et al. IMP3 overexpression occurs in various important cancer types and is linked to aggressive tumor features: A tissue microarray study on 8,877 human cancers and normal tissues. *Oncol Rep*. 2018;39(1):3–12. doi: 10.3892/or.2017.6072 29115542 PMC5783598

[14] Chiang K, Zielinska AE, Shaaban AM, Sanchez-Bailon MP, Jarrold J, Clarke TL, et al. PRMT5 Is a Critical Regulator of Breast Cancer Stem Cell Function via Histone Methylation and FOXP1 Expression. *Cell Rep*. 2017;21(12):3498–3513. doi: 10.1016/j.celrep.2017.11.096 29262329 PMC5746596

[15] Brown P, Marafioti T, Kusec R, Banham AH. The FOXP1 transcription factor is expressed in the majority of follicular lymphomas but is rarely expressed in classical and lymphocyte predominant Hodgkin’s lymphoma. *J Mol Histol*. 2005;36(4):249–56. doi: 10.1007/s10735-005-6521-3 16200457

[16] Hoefnagel JJ, Mulder MM, Dreef E, Jansen PM, Pals ST, Meijer CJ, et al. Expression of B-cell transcription factors in primary cutaneous B-cell lymphoma. *Mod Pathol*. 2006;19(9):1270–6. doi: 10.1038/modpathol.3800650 16778825

[17] Espinet B, Garcia-Herrera A, Gallardo F, Baro C, Salgado R, Servitje O, et al. FOXP1 molecular cytogenetics and protein expression analyses in primary cutaneous large B cell lymphoma, leg-type. *Histol Histopathol*. 2011;26(2):213–21. doi: 10.14670/HH-26.213 21154235

[18] Wlodarska I, Veyt E, De Paepe P, Vandenberghe P, Nooijen P, Theate I, et al. FOXP1, a gene highly expressed in a subset of diffuse large B-cell lymphoma, is recurrently targeted by genomic aberrations. *Leukemia*. 2005;19(8):1299–305. doi: 10.1038/sj.leu.2403813 15944719

[19] Banham AH, Connors JM, Brown PJ, Cordell JL, Ott G, Sreenivasan G, et al. Expression of the FOXP1 transcription factor is strongly associated with inferior survival in patients with diffuse large B-cell lymphoma. *Clin Cancer Res*. 2005;11(3):1065–72. 15709173

[20] Sagaert X, de Paepe P, Libbrecht L, Vanhentenrijk V, Verhoef G, Thomas J, et al. Forkhead box protein P1 expression in mucosa-associated lymphoid tissue lymphomas predicts poor prognosis and transformation to diffuse large B-cell lymphoma. *J Clin Oncol*. 2006;24(16):2490–7. doi: 10.1200/JCO.2006.05.6150 16636337

[21] Wang X, Sun J, Cui M, Zhao F, Ge C, Chen T, et al. Downregulation of FOXP1 Inhibits Cell Proliferation in Hepatocellular Carcinoma by Inducing G1/S Phase Cell Cycle Arrest. *Int J Mol Sci*. 2016;17(9):1501. doi: 10.3390/ijms17091501 27618020 PMC5037778

[22] Brown PJ, Wong KK, Felce SL, Lyne L, Spearman H, Soilleux EJ, et al. FOXP1 suppresses immune response signatures and MHC class II expression in activated B-cell-like diffuse large B-cell lymphomas. *Leukemia*. 2016;30(3):605–16. doi: 10.1038/leu.2015.299 26500140 PMC4777777

[23] Walker MP, Stopford CM, Cederlund M, Fang F, Jahn C, Rabinowitz AD, et al. FOXP1 potentiates Wnt/beta-catenin signaling in diffuse large B cell lymphoma. *Sci Signal*. 2015;8(362):ra12. doi: 10.1126/scisignal.2005654 25650440 PMC4356208

[24] Bates GJ, Fox SB, Han C, Launchbury R, Leek RD, Harris AL, et al. Expression of the forkhead transcription factor FOXP1 is associated with that of estrogen receptor-beta in primary invasive breast carcinomas. *Breast Cancer Res Treat*. 2008;111(3):453–9. doi: 10.1007/s10549-007-9812-4 18026833

[25] Halacli SO, Dogan AL. FOXP1 regulation via the PI3K/Akt/p70S6K signaling pathway in breast cancer cells. *Oncol Lett*. 2015;9(3):1482–1488. doi: 10.3892/ol.2015.2885 25663935 PMC4315073

[26] Sheng H, Li X, Xu Y. Knockdown of FOXP1 promotes the development of lung adenocarcinoma. *Cancer Biol Ther*. 2019;20(4):537–45. doi: 10.1080/15384047.2018.1537999 30409062 PMC6422458

[27] Takayama K, Suzuki T, Tsutsumi S, Fujimura T, Takahashi S, Homma Y, et al. Integrative analysis of FOXP1 function reveals a tumor-suppressive effect in prostate cancer. *Mol Endocrinol*. 2014;28(12):2012–24. doi: 10.1210/me.2014-1171 25329375 PMC5414778

[28] Oskay Halacli S. FOXP1 enhances tumor cell migration by repression of NFAT1 transcriptional activity in MDA-MB-231 cells. *Cell Biol Int*. 2017;41(1):102–110. doi: 10.1002/cbin.10702 27859969

[29] Qiu DP, Han F, Zhuang H, Li QJ, Zhang XZ. Overexpression of FoxP1 is a novel biomarker of malignant human pancreatic cancer. *Int J Clin Exp Med*. 2016, 9(6):9054–9063.

[30] Banham AH, Beasley N, Campo E, Fernandez PL, Fidler C, Gatter K, et al. The FOXP1 winged helix transcription factor is a novel candidate tumor suppressor gene on chromosome 3p. *Cancer Res*. 2001;61(24):8820–9. 11751404

[31] Lim R, Tran HT, Liong S, Barker G, Lappas M. The Transcription Factor Interferon Regulatory Factor-1 (IRF1) Plays a Key Role in the Terminal Effector Pathways of Human Preterm Labor. *Biol Reprod*. 2016;94(2):32. doi: 10.1095/biolreprod.115.134726 26674566

[32] Zhan FB, Liu H, Lai RF, Jakovlic I, Wang WB, Wang WM. Molecular identification and functional characterisation of the interferon regulatory factor 1 in the blunt snout bream (Megalobrama amblycephala). *Fish Shellfish Immunol*. 2016;54:456–65. doi: 10.1016/j.fsi.2016.05.002 27150048

[33] Shah S, King EM, Mostafa MM, Altonsy MO, Newton R. DUSP1 Maintains IRF1 and Leads to Increased Expression of IRF1-dependent Genes: A MECHANISM PROMOTING GLUCOCORTICOID INSENSITIVITY. *J Biol Chem*. 2016;291(41):21802–21816. doi: 10.1074/jbc.M116.728964 27551049 PMC5076847

[34] Lorenzi S, Forloni M, Cifaldi L, Antonucci C, Citti A, Boldrini R, et al. IRF1 and NF-kB restore MHC class I-restricted tumor antigen processing and presentation to cytotoxic T cells in aggressive neuroblastoma. *PloS One*. 2012;7(10):e46928. doi: 10.1371/journal.pone.0046928 23071666 PMC3465322

[35] Walch-Ruckheim B, Pahne-Zeppenfeld J, Fischbach J, Wickenhauser C, Horn LC, Tharun L, et al. STAT3/IRF1 Pathway Activation Sensitizes Cervical Cancer Cells to Chemotherapeutic Drugs. *Cancer Res*. 2016;76(13):3872–83. doi: 10.1158/0008-5472.CAN-14-1306 27216197

[36] Xie C, Liu C, Wu B, Lin Y, Ma T, Xiong H, et al. Effects of IRF1 and IFN-beta interaction on the M1 polarization of macrophages and its antitumor function. *Int J Mol Med*. 2016;38(1):148–60. doi: 10.3892/ijmm.2016.2583 27176664 PMC4899022

