# Supplementary figures and images for: FOXP1 inhibits pancreatic cancer growth by transcriptionally regulating IRF1 expression
Source: PLoS One. 2023 Mar 23;18(3):e0280794. doi: 10.1371/journal.pone.0280794 (PMC10035899; doi:10.1371/journal.pone.0280794)

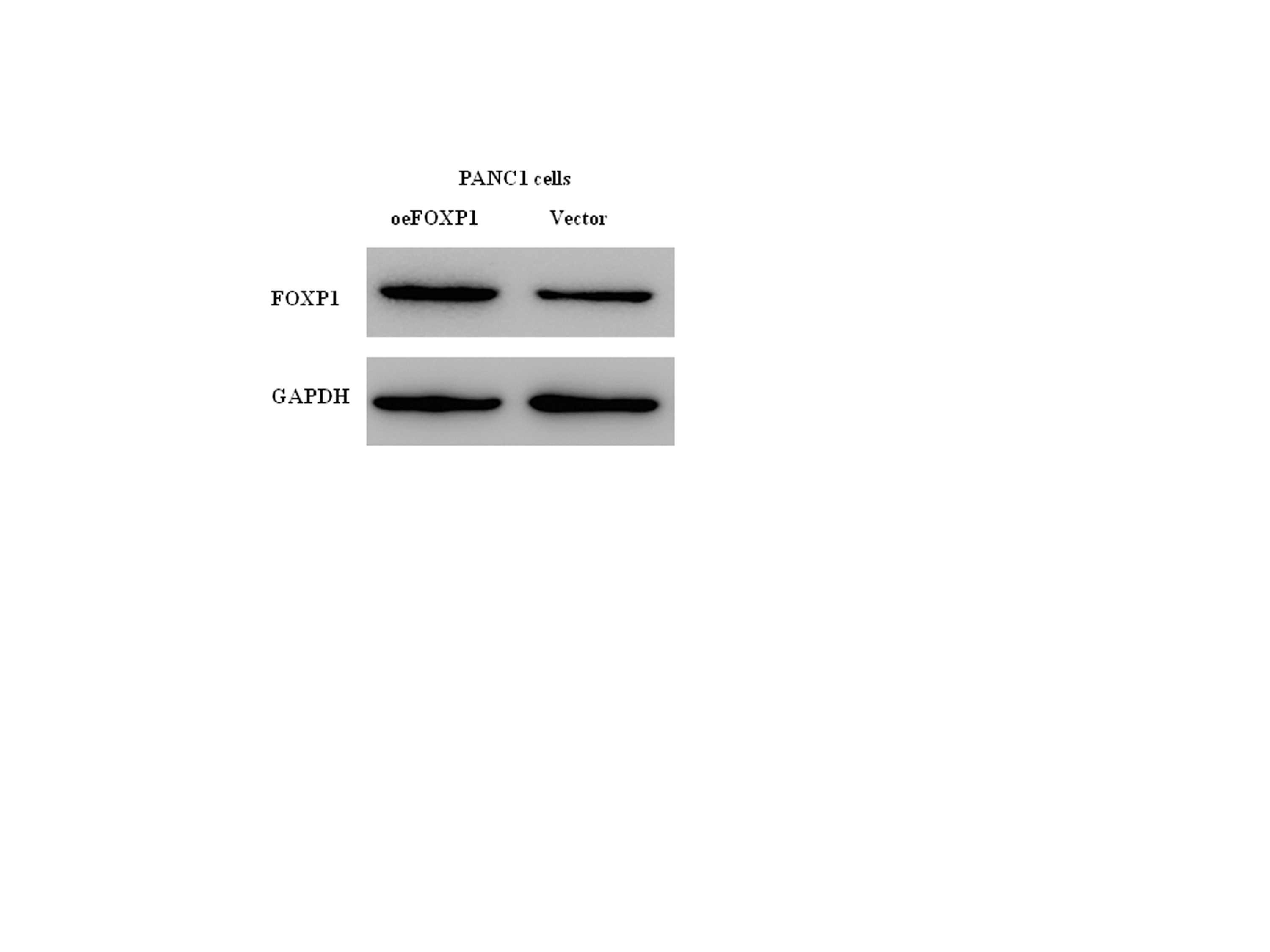

Supplement: S1 Fig — (TIF) [file pone.0280794.s001.TIF]
