# Supplementary material for: FOXP1 inhibits pancreatic cancer growth by transcriptionally regulating IRF1 expression
Source: PLoS One. 2023 Mar 23;18(3):e0280794. doi: 10.1371/journal.pone.0280794 (PMC10035899; doi:10.1371/journal.pone.0280794)
Supplement: S2 Table — (DOC) [file pone.0280794.s003.doc]

**Supplementary Table 2. mutIRF1Promoter**

TGCCCTGGAGGGAGAGCTGGGGTGAAGGAAATGACACGCCTGGGAGAGTAACTTACTTCTGCAGGAGCTTTAGGGAGATGAAGGAAGAAGCCTCCTGGGCCAGAGTTTTGGATGGAAAATGAACACCCAGTCAAGTCTCTAGGACTATACGTGGGGCGGGGACTAGTTGTGCGCGAGAGTTAAGTAGGGGCCTTACCAAGGAGCATGGGACCTGGGCTCCCCAACCCTTTGGCTAGCCCCATGGCGTTGATCAGCCCTGAGCTAATTCCTCCATGCTGCCCAGAACCTCTCTGGGCCAAGCCCTGGGGACTCAGAGATGACAGCAATGCTTCCATTGCGGAACTCCCATACGCGGGCCACAGGGAGGCTCTGGAGGCGGCCTGAGGCAAGAGTGCTAGGAGGGATCAGAGCTAGCCCACCCCTACCCTCACTCAGCCGTCTGGGCTTCTCTGAACCCCTTCTCCTCCTCTGTTCCCTAAAGCCAGCCAGGGGGAGTCCCAGGGAGGCAGACCGAAAAGGGGTGGGGTGTCATCCTGGTCACTATTAGACCCTGCAACGGCGACCTTGAAAACTACTCAGCGTCTGTTGCCCGAGTGGAGCATAGTGCTTTACAATCTCTTCCCATCACAGCAAACCATCAAGGTAGGGCTACTATTATTTTATATCCTCGGCGTCTCAGTCCTGCGTCCCTTGGGGGCTGTGCCAGCAGCGGCCAAGTTGGGATTTCCCCTGGTCCAGCAGCCCCAGACAGCACACGGGGCAGGGTAGGCTTTCTGCCTTCTTCACTTCCCCAGGGCAGGTGAGTGACCTGGAGGGAGGGGGTCACCCTGGGCCGTCAATGTAGTGCTAGGACTGAAACCCTCCCTTCTTGATATCCCACTGGCAAGCTTGAGGAGCCAGGCTGCCAGTCGGGAGATTCGGCCCAGTGTTCCCACTGGAGAGGGCGGCAAGTGCCCGGGCGATCCCCTCACCTGCGTTCGGGAGATATACCCCCGCCCCCGCCCCGCCAGGAGGGTGAAAAGATGGCCCCAGGAGCCAGCCGGCTGGGACAAGGCGGAGTGAGAGGACAGGCTGGGGCCAGGGGCGCTGGGCTGTCCCGGGCAGCCCTCCTCCGGGCAAGCCGGAGCAGGGGTGGATTGGGAGCGCTCGGGGCGGGCCCGCGGTGGCCCCGGGGCGGTGGCGCCCGGCCGGAGAGGGTGGGGCGGAGCAGCCGCCCTGTACTTCCCCTTCGCCGCTAGCTCTACAACAGCCTGATTTCCCCGAAATGACGGCACGCAGCCGGCCAATGGGCGCCCGCGCGGCTGTCCGGGGGCGGGGCCGGCCAGGGCTGGGGAATCCCGCTAAGTGTTTGGATTGCTCGGTGGCGCCGCTGCCCTGGC**A**GAGCTCGCCACTCCTTAGTCGAGGCAAGACGTGCGCCCGAGCCCCGCCGAACCGAGGCCACCCGGAGCCGTGCCCAGTCCACGCCGGCCGTGCCCGGCGGCCTTAAGAACCCGGCAACCTCTGCCTTCTTCCCTCTTCCACTCGGAGTCGCGCTCCGCGCGCCCTCACTGCAGCCCCTGCGT

Note: Binding-site mutation (blue).
