# Supplementary material for: FOXP1 inhibits pancreatic cancer growth by transcriptionally regulating IRF1 expression
Source: PLoS One. 2023 Mar 23;18(3):e0280794. doi: 10.1371/journal.pone.0280794 (PMC10035899; doi:10.1371/journal.pone.0280794)
Supplement: S1 File — (DOCX) [file pone.0280794.s004.docx]

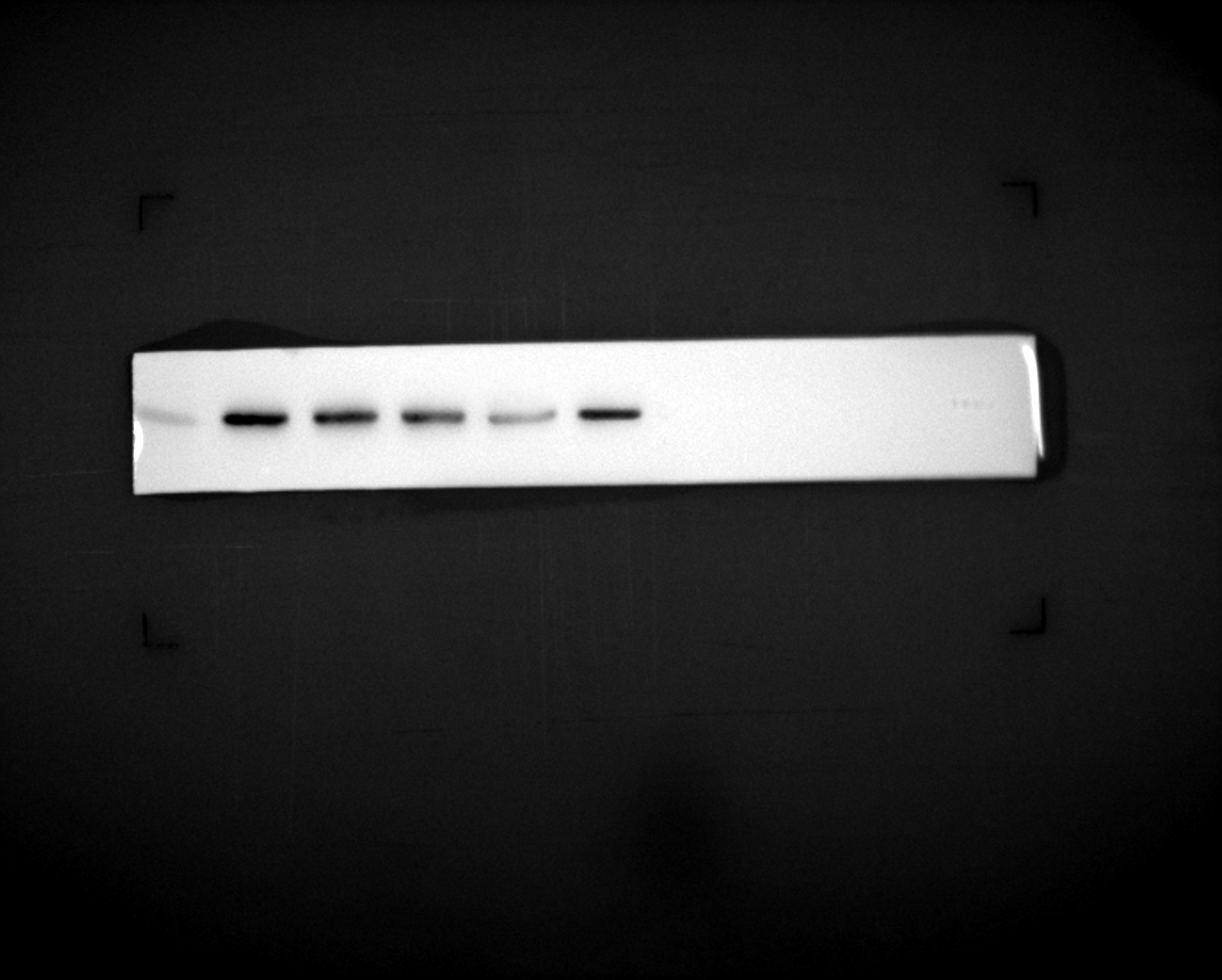

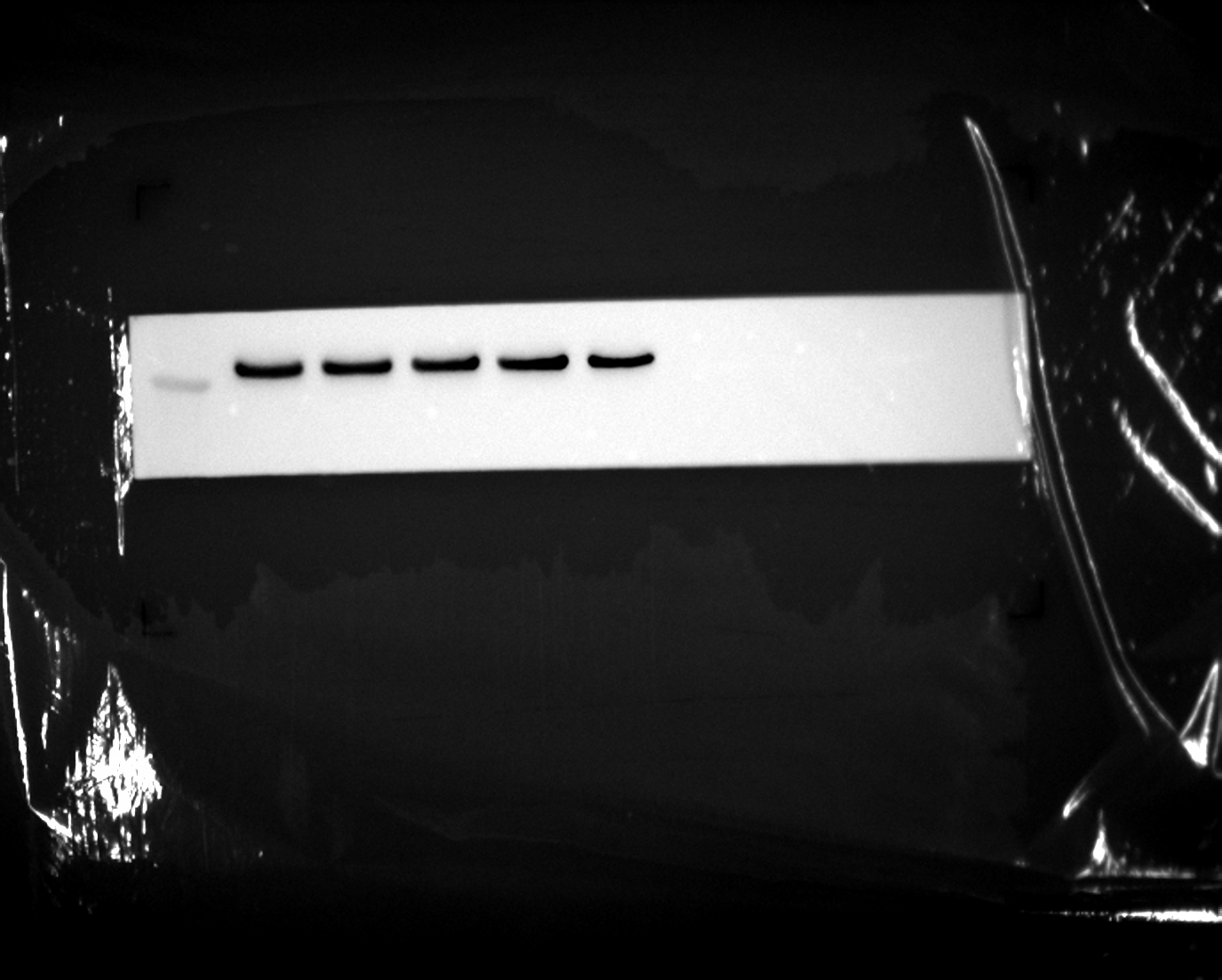


Fig 3A FOXP1 Fig 3A GAPDH


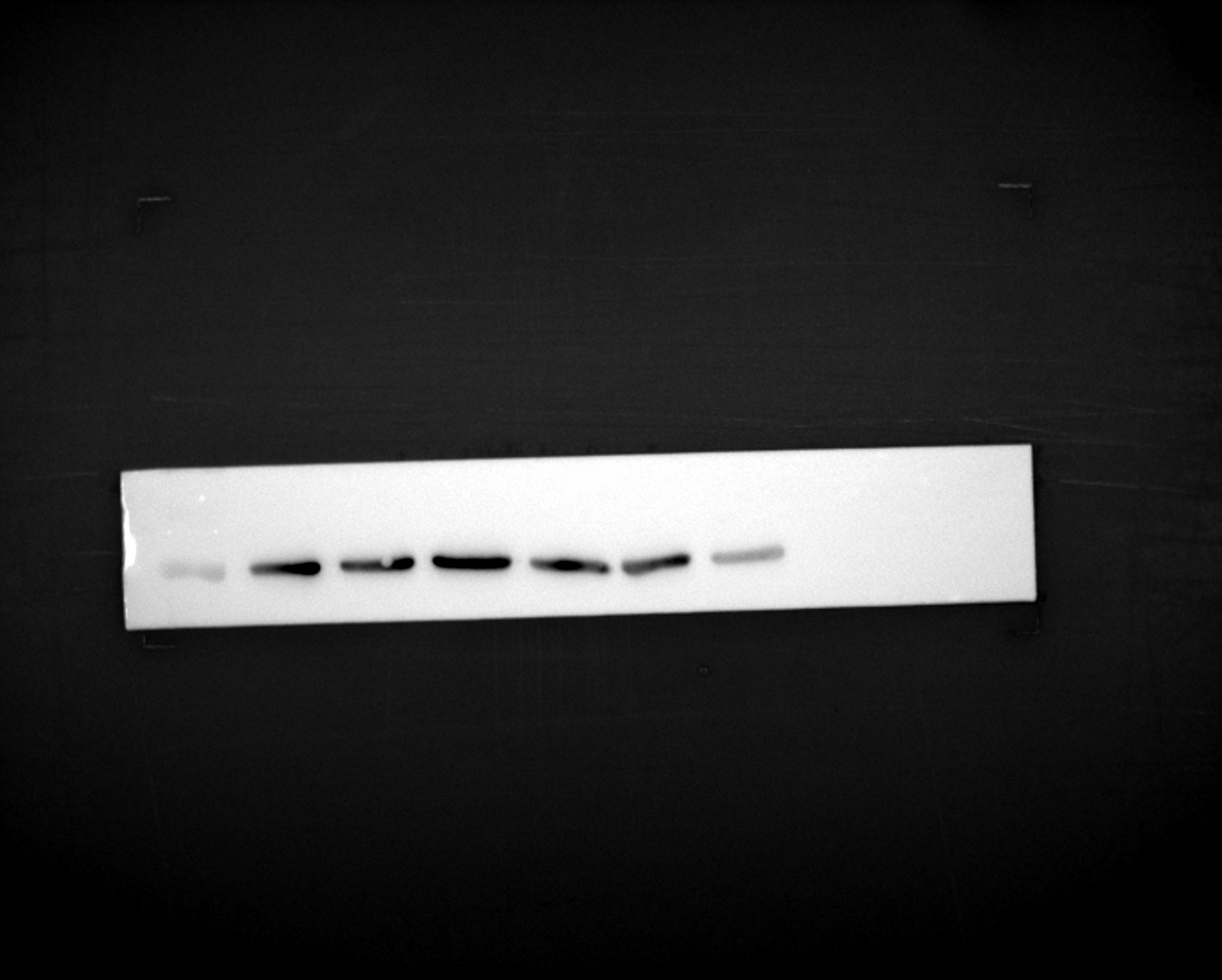

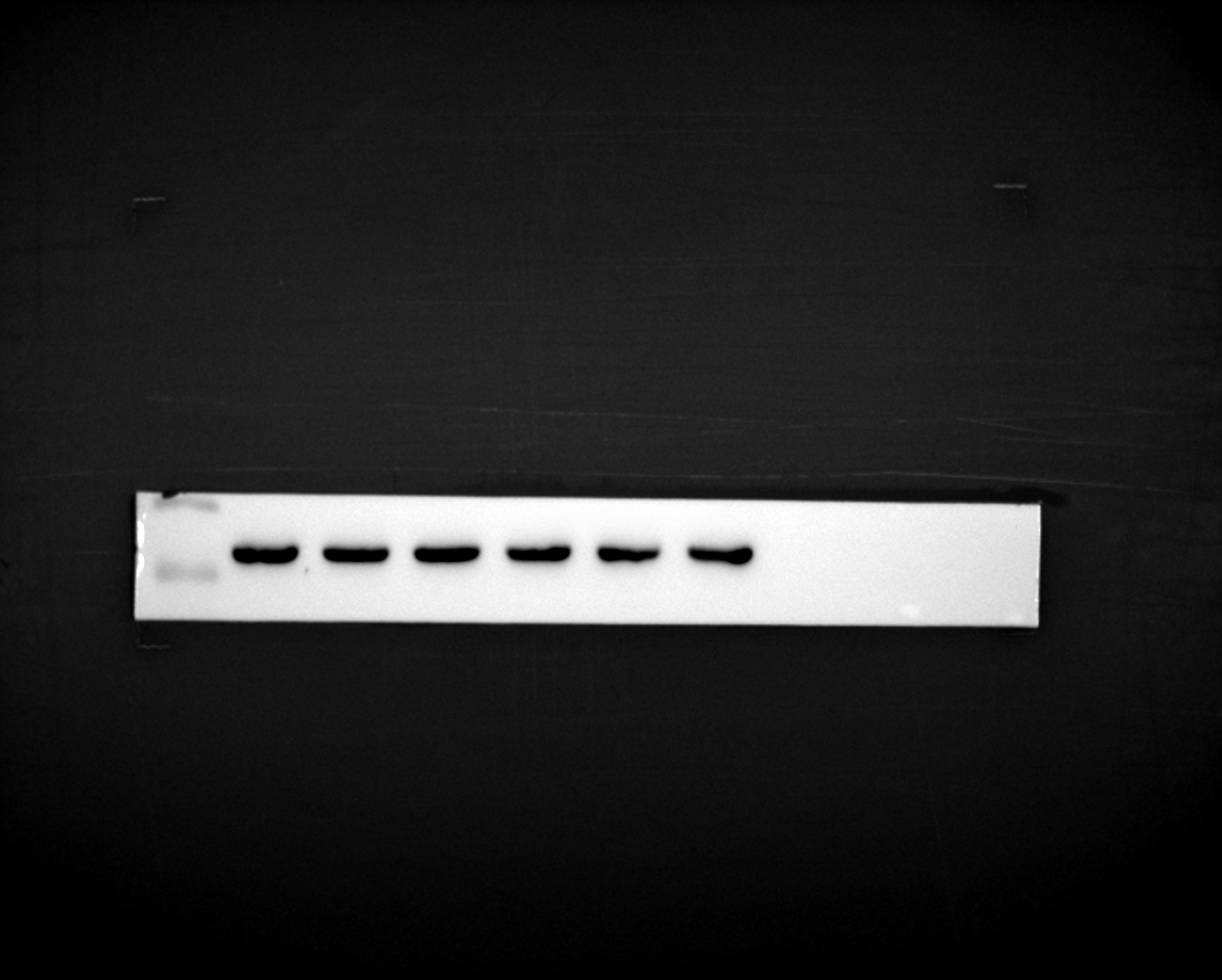


Fig 3B FOXP1 Fig 3B GAPDH


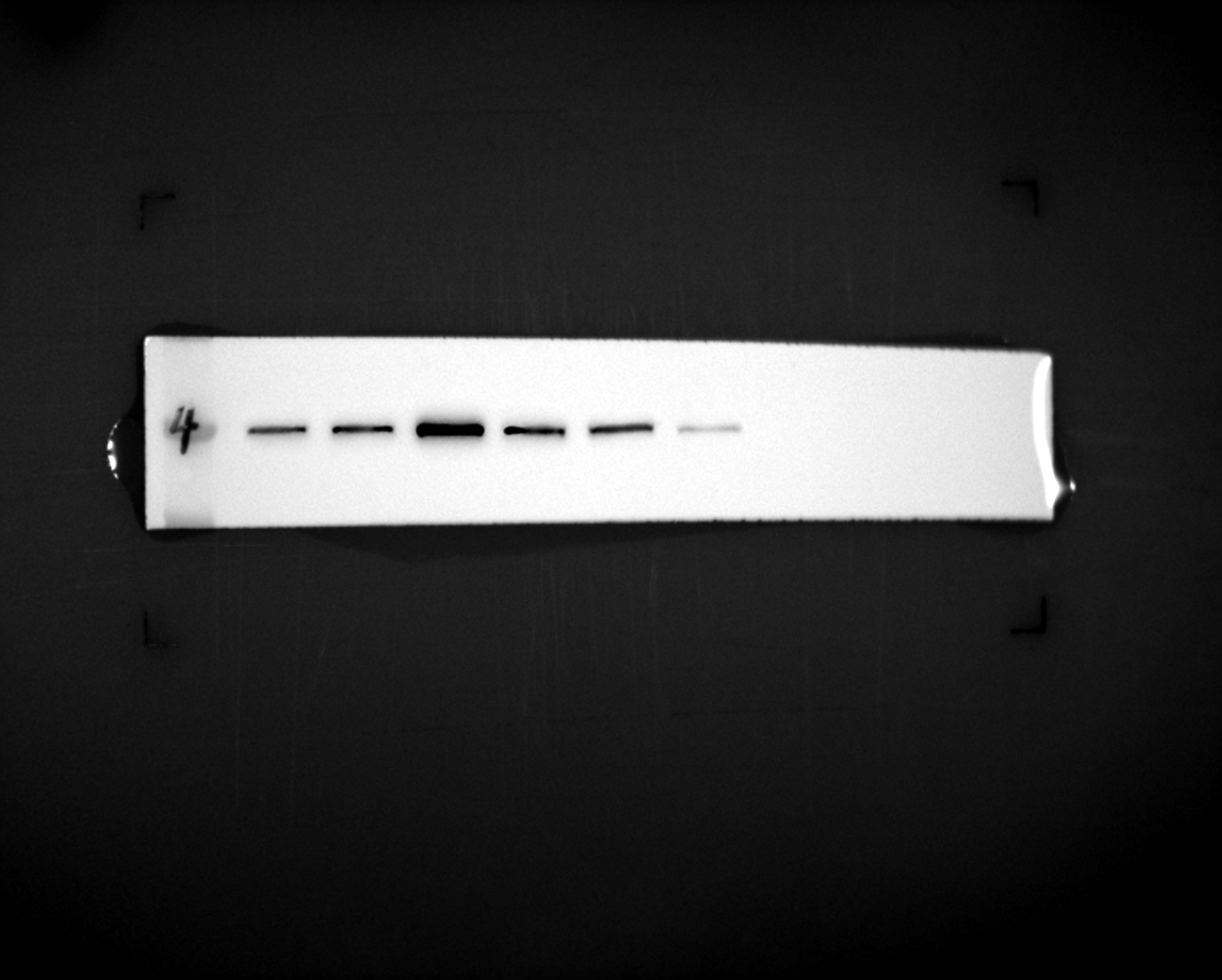

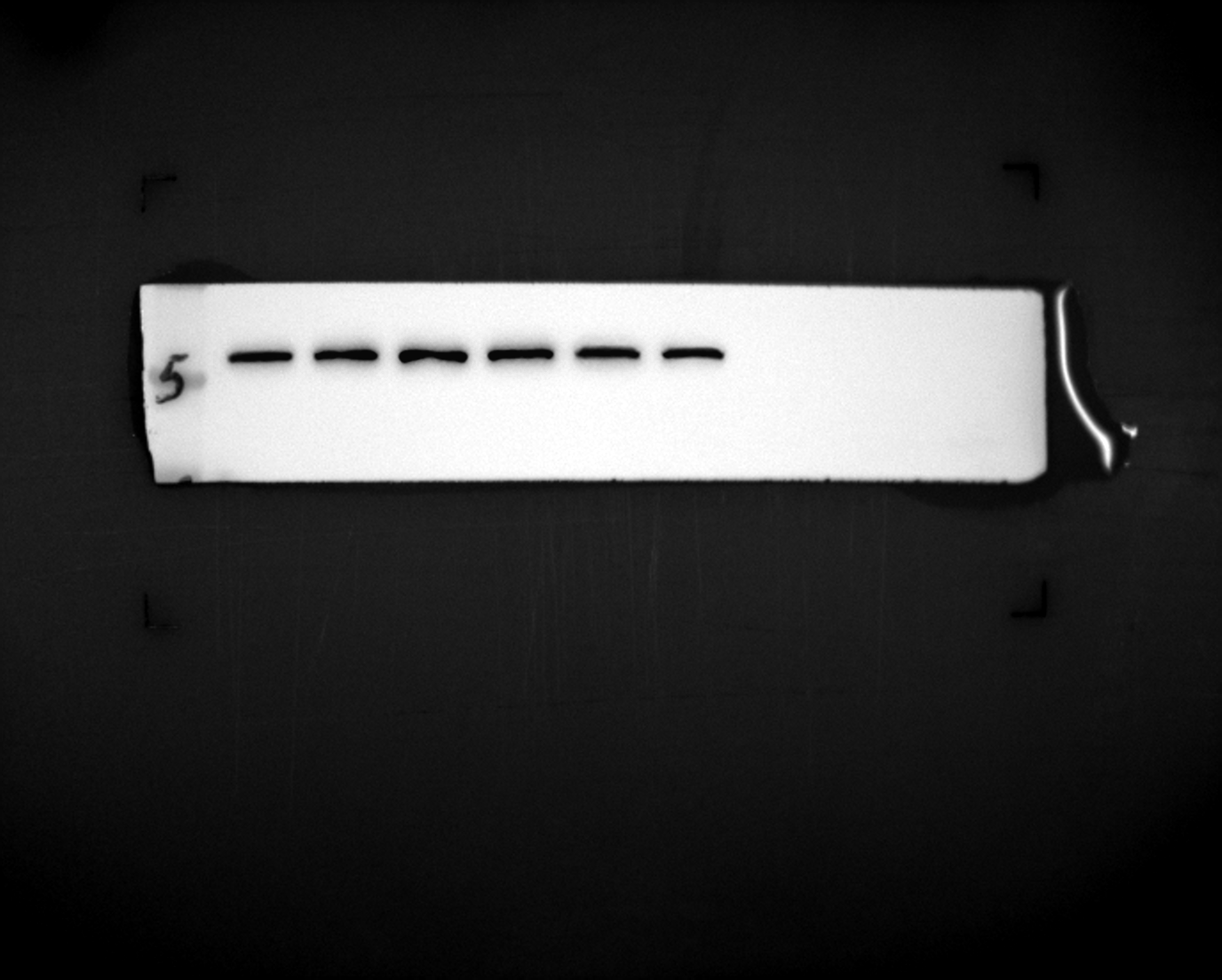


Fig 3 C FOXP1 Fig 3C GAPDH


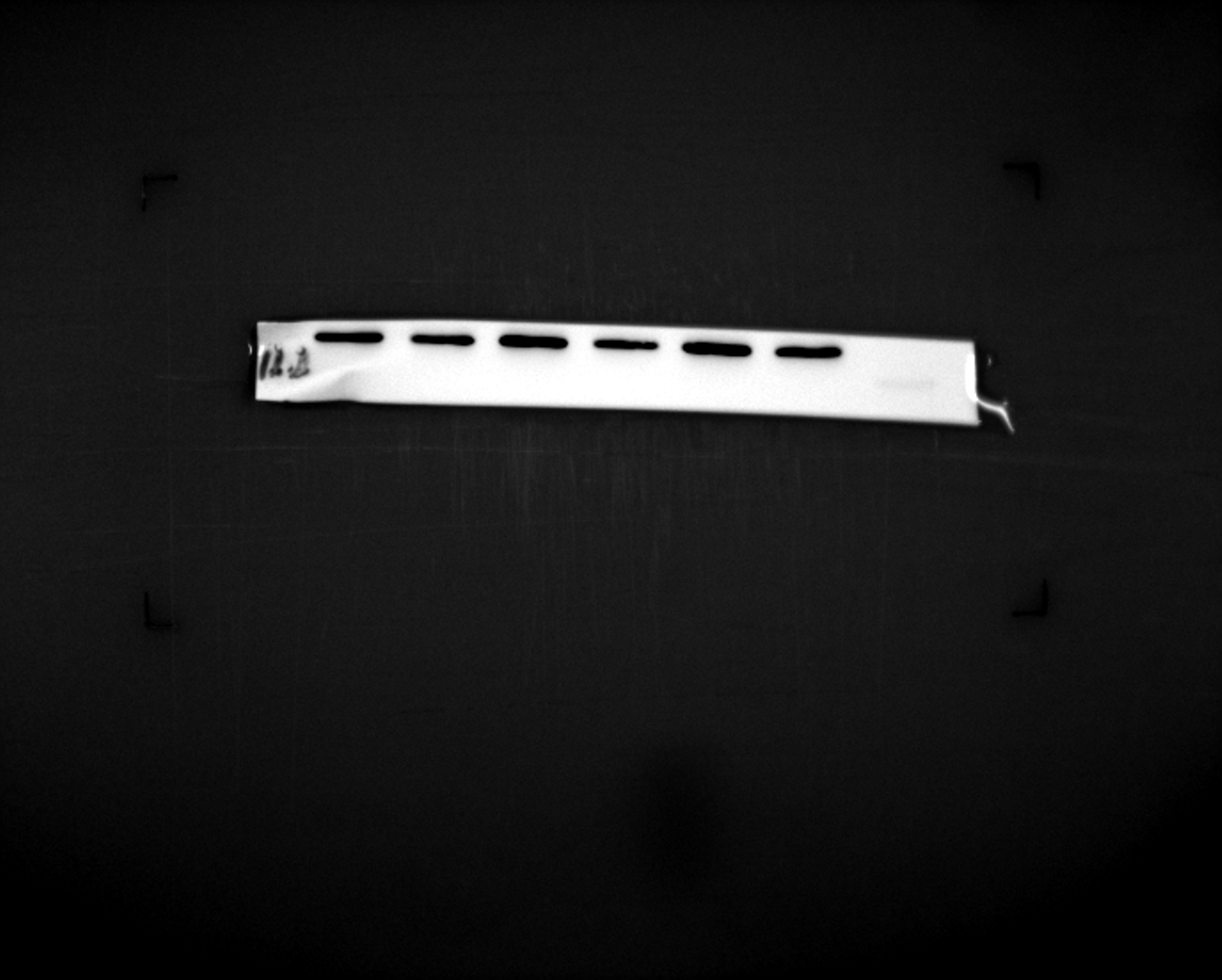

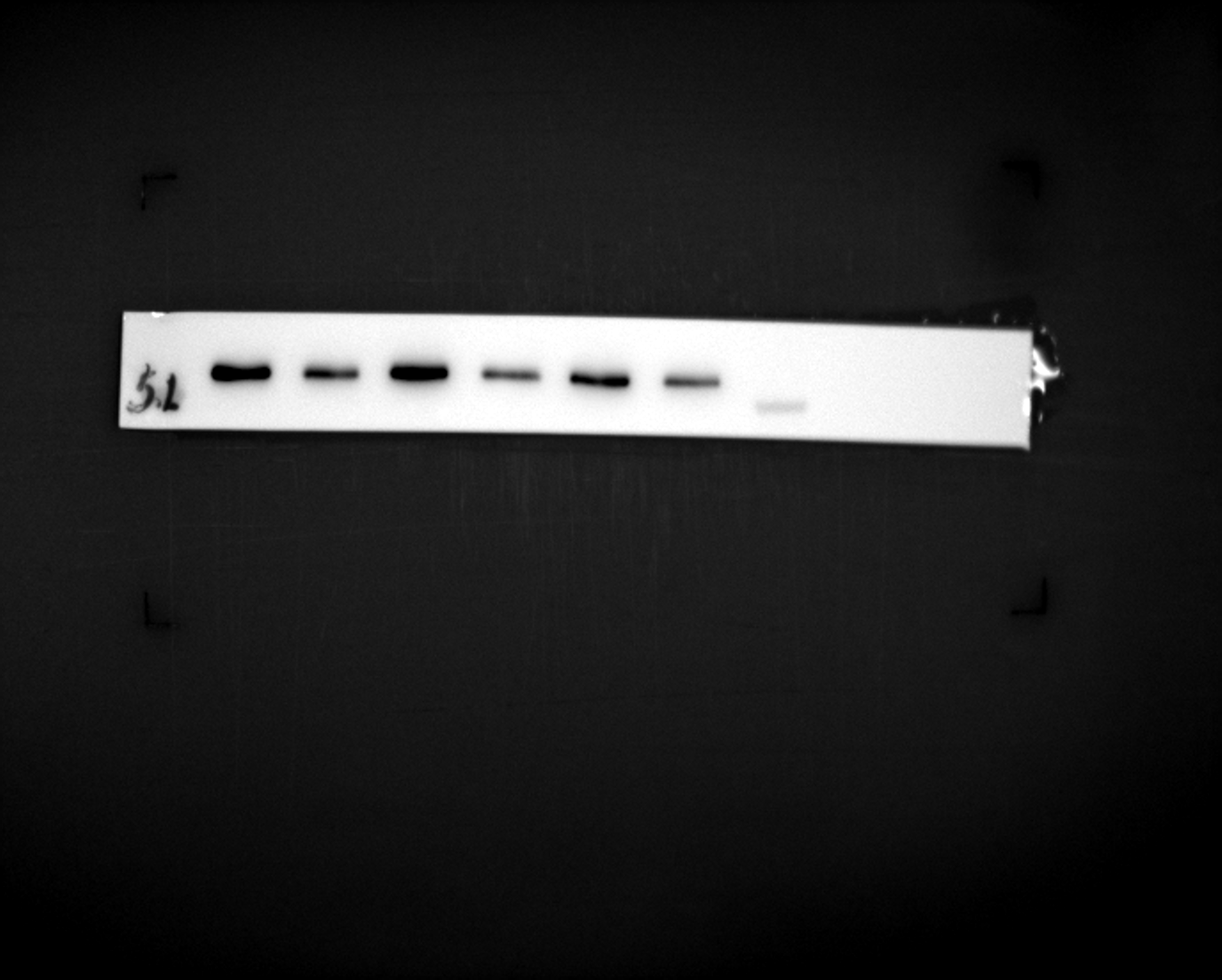


Fig 6 B GAPDH Fig 6 B IRF1


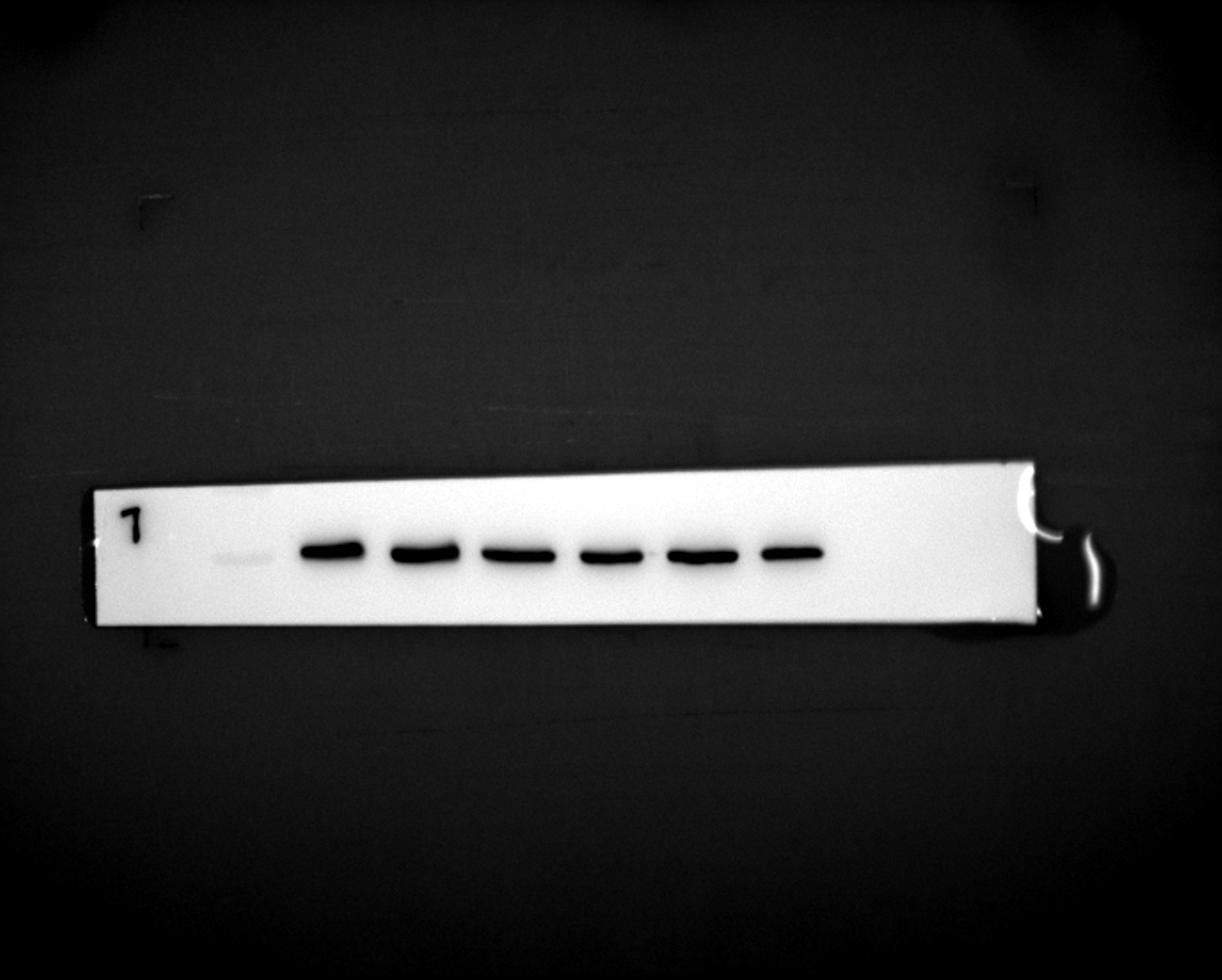

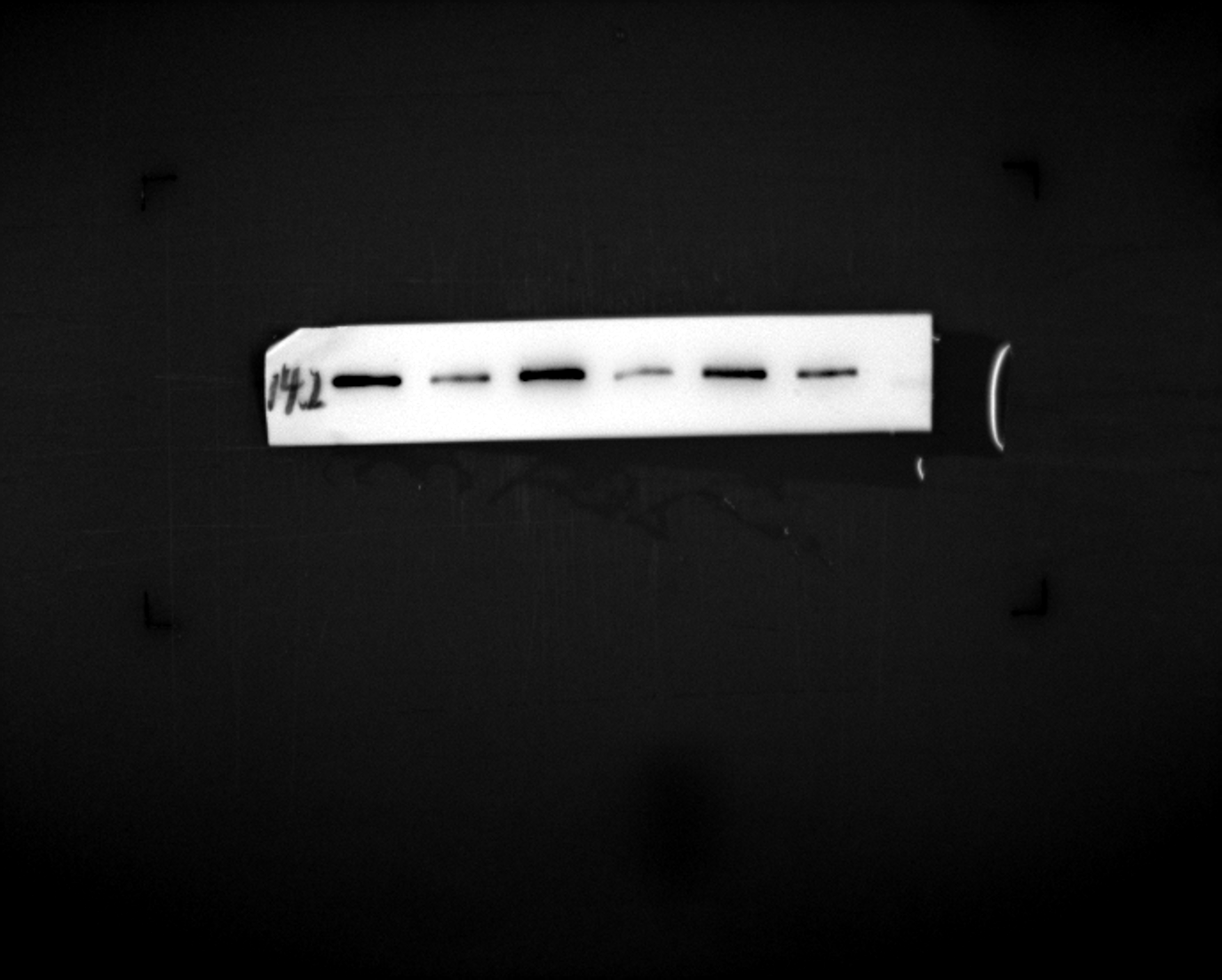


Fig 6 C GAPDH Fig 6 C IRF1


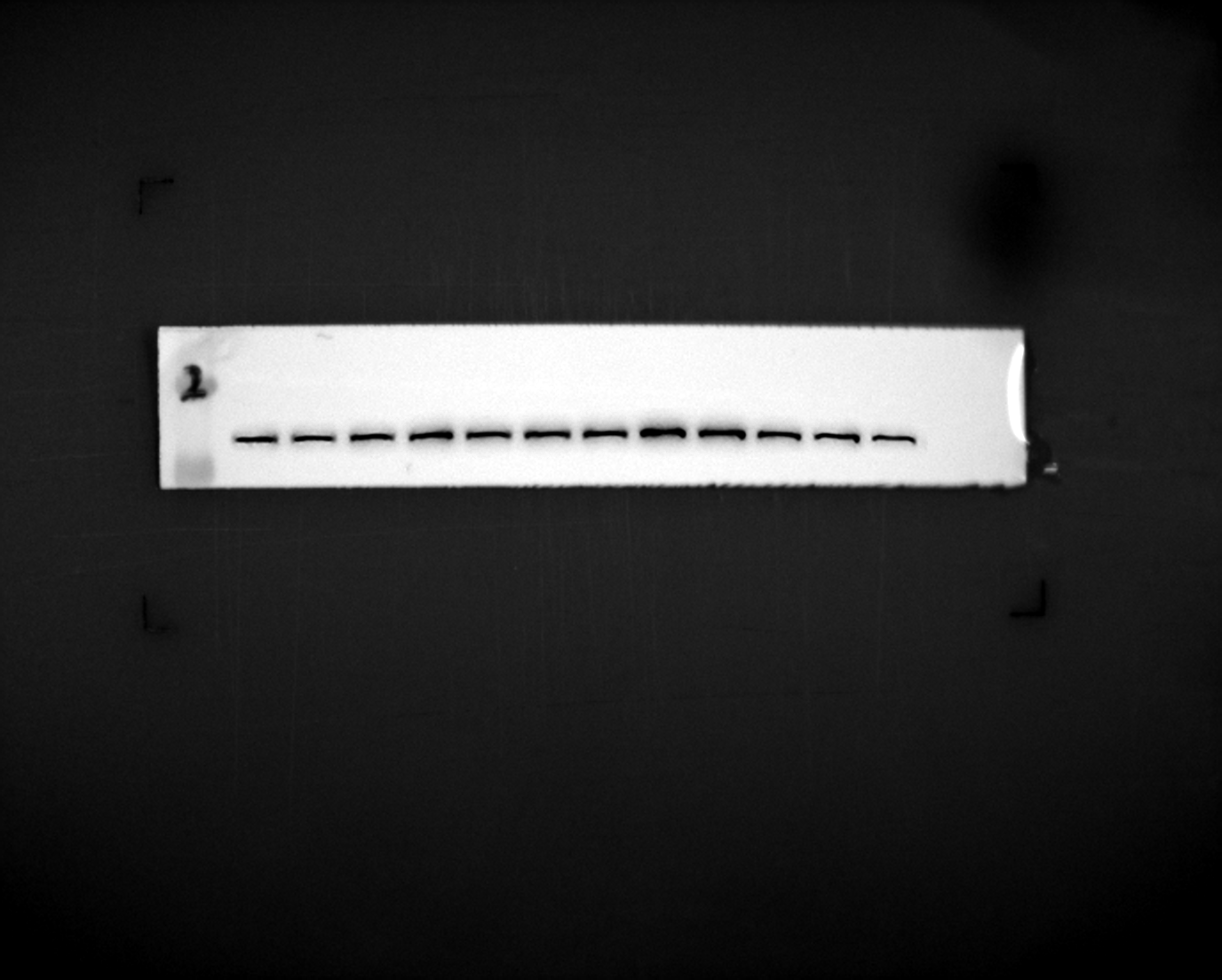

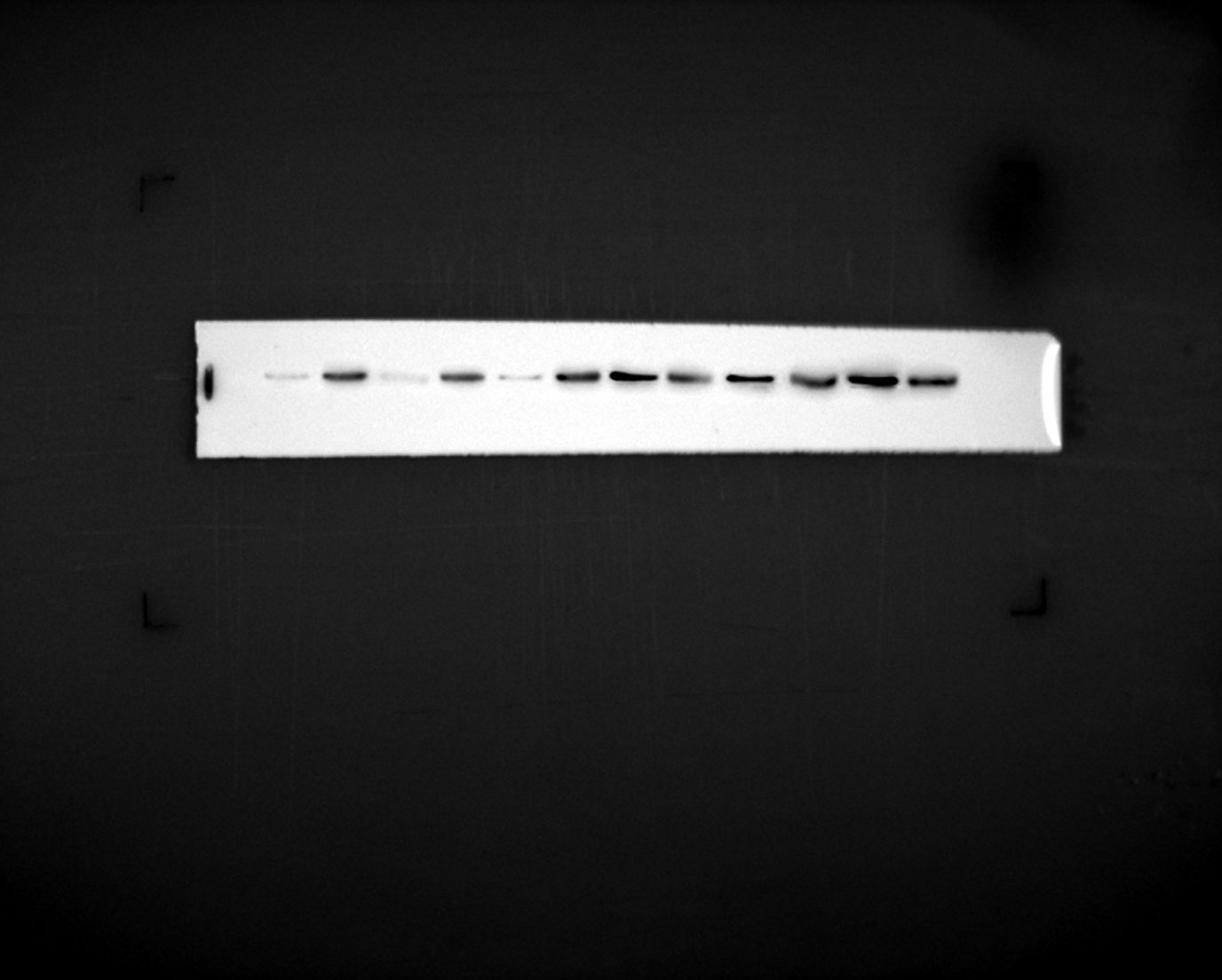


Fig 6 D GAPDH Fig 6 D IRF1


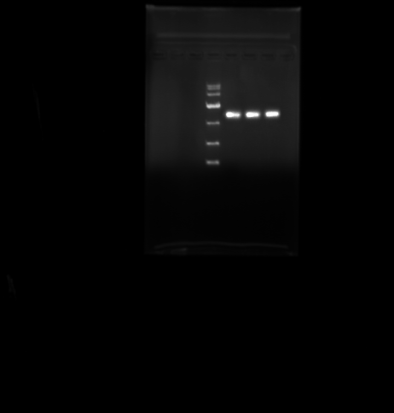

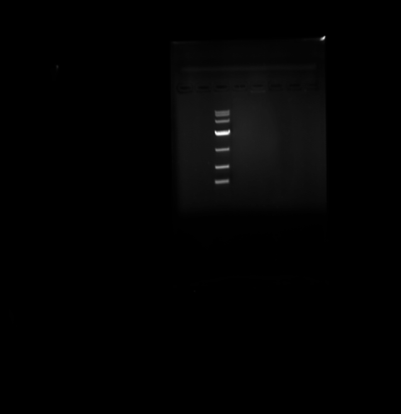

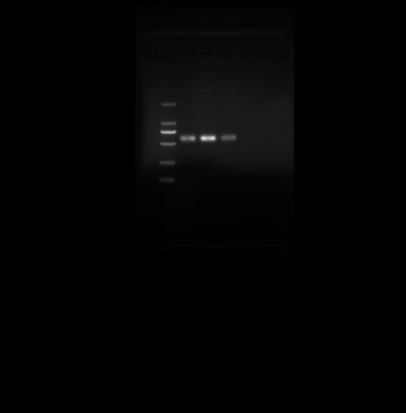


FOXP1 IgG Input


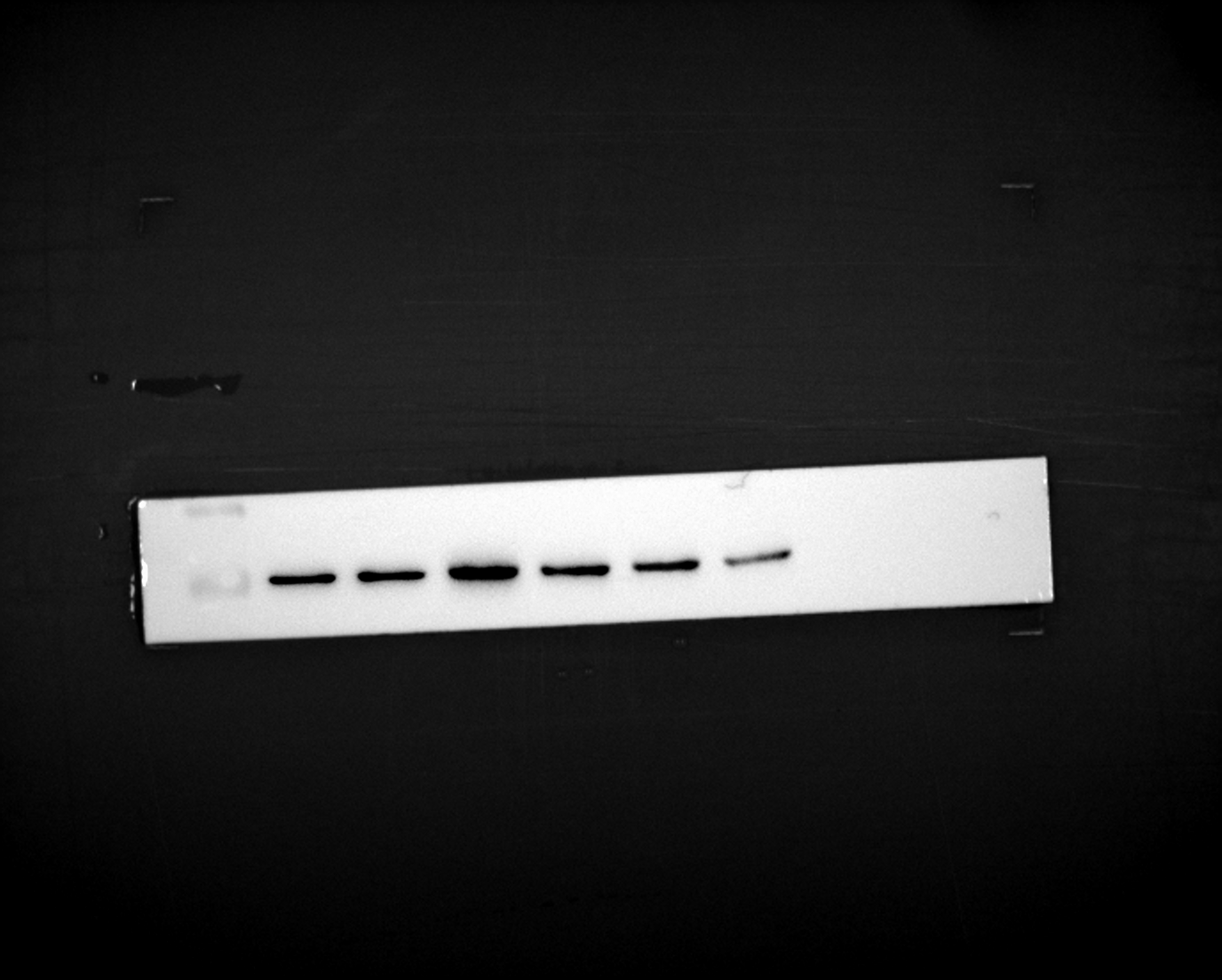

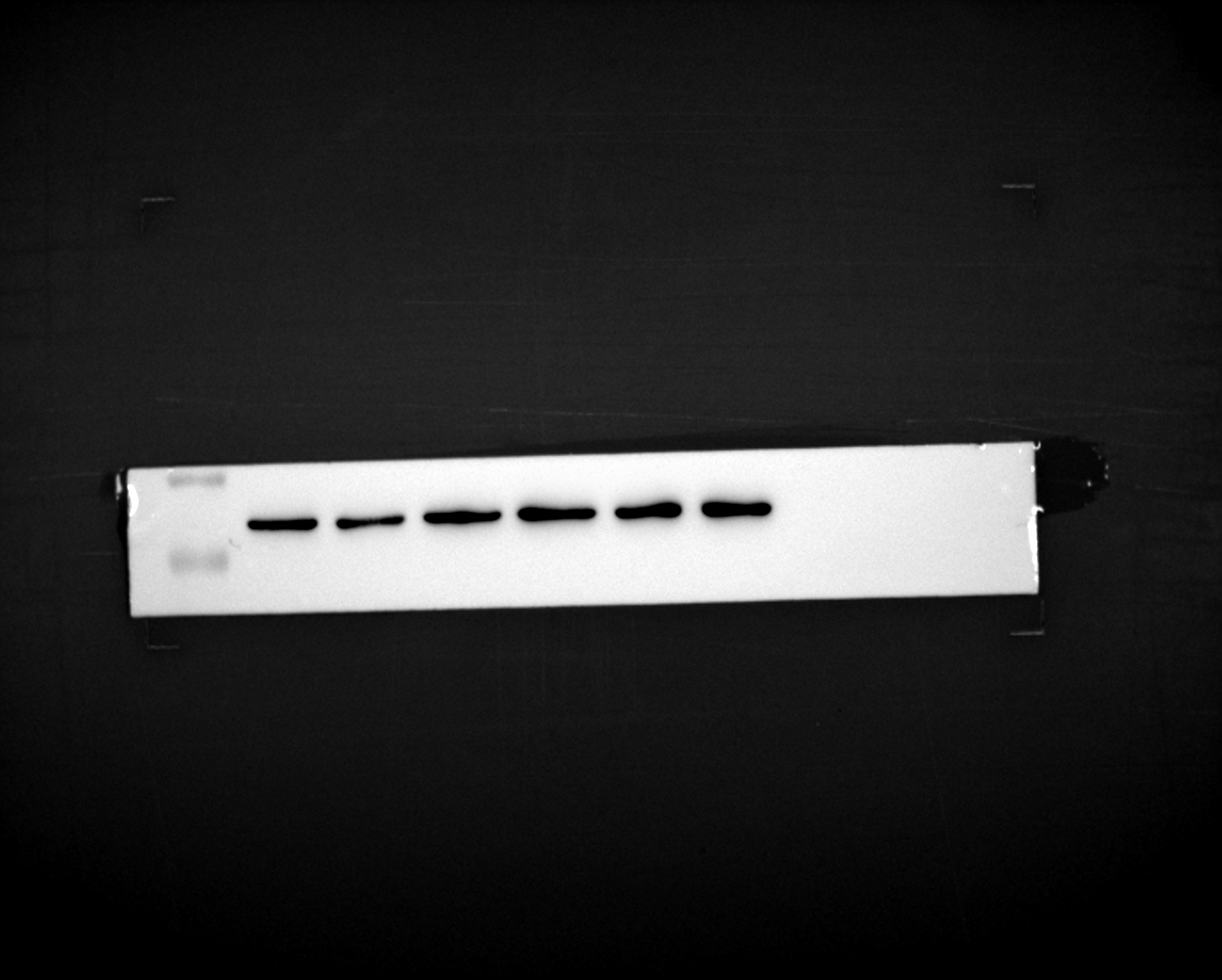


S1 Fig FOXP1 S1 Fig GAPDH
